# Reference-Grade Genome and Large Linear Plasmid of Streptomyces rimosus: Pushing the Limits of Nanopore Sequencing

**DOI:** 10.1128/spectrum.02434-21

**Published:** 2022-04-04

**Authors:** Lucija Slemc, Jernej Jakše, Alessandro Filisetti, Damir Baranasic, Antonio Rodríguez-García, Francesco Del Carratore, Stefano Maria Marino, Jurica Zucko, Antonio Starcevic, Martin Šala, Mercedes Pérez-Bonilla, Marina Sánchez-Hidalgo, Ignacio González, Fernando Reyes, Olga Genilloud, Vicki Springthorpe, Dušan Goranovič, Gregor Kosec, Gavin H. Thomas, Davide De Lucrezia, Hrvoje Petković, Miha Tome

**Affiliations:** a Food Science and Technology Department, Biotechnical Faculty, University of Ljubljana, Ljubljana, Slovenia; b Department of Agronomy, Biotechnical Faculty, University of Ljubljana, Ljubljana, Slovenia; c Explora Biotech Srl, Venice, Italy; d Faculty of Food Technology and Biotechnology, University of Zagreb, Zagreb, Croatia; e INBIOTEC Instituto de Biotecnología de León, Parque Científico de la Granja, León, Spain; f Manchester Institute of Biotechnology, Faculty of Science and Engineering, University of Manchester, Manchester, United Kingdom; g National Institute of Chemistry, Ljubljana, Slovenia; h Fundación MEDINA, Parque Tecnológico Ciencias de la Salud, Granada, Spain; i Department of Biology, University of York, York, United Kingdom; j Acies Bio d.o.o., Ljubljana, Slovenia; Washington University in St. Louis

**Keywords:** Oxford Nanopore sequencing, *Streptomyces rimosus*, biosynthetic gene clusters, genome, linear plasmid, oxytetracycline, pulsed-field electrophoresis, transposase

## Abstract

Streptomyces rimosus ATCC 10970 is the parental strain of industrial strains used for the commercial production of the important antibiotic oxytetracycline. As an actinobacterium with a large linear chromosome containing numerous long repeat regions, high GC content, and a single giant linear plasmid (GLP), these genomes are challenging to assemble. Here, we apply a hybrid sequencing approach relying on the combination of short- and long-read next-generation sequencing platforms and whole-genome restriction analysis by using pulsed-field gel electrophoresis (PFGE) to produce a high-quality reference genome for this biotechnologically important bacterium. By using PFGE to separate and isolate plasmid DNA from chromosomal DNA, we successfully sequenced the GLP using Nanopore data alone. Using this approach, we compared the sequence of GLP in the parent strain ATCC 10970 with those found in two semi-industrial progenitor strains, R6-500 and M4018. Sequencing of the GLP of these three *S. rimosus* strains shed light on several rearrangements accompanied by transposase genes, suggesting that transposases play an important role in plasmid and genome plasticity in *S. rimosus*. The polished annotation of secondary metabolite biosynthetic pathways compared to metabolite analysis in the ATCC 10970 strain also refined our knowledge of the secondary metabolite arsenal of these strains. The proposed methodology is highly applicable to a variety of sequencing projects, as evidenced by the reliable assemblies obtained.

**IMPORTANCE** The genomes of *Streptomyces* species are difficult to assemble due to long repeats, extrachromosomal elements (giant linear plasmids [GLPs]), rearrangements, and high GC content. To improve the quality of the *S. rimosus* ATCC 10970 genome, producer of oxytetracycline, we validated the assembly of GLPs by applying a new approach to combine pulsed-field gel electrophoresis separation and GLP isolation and sequenced the isolated GLP with Oxford Nanopore technology. By examining the sequenced plasmids of ATCC 10970 and two industrial progenitor strains, R6-500 and M4018, we identified large GLP rearrangements. Analysis of the assembled plasmid sequences shed light on the role of transposases in genome plasticity of this species. The new methodological approach developed for Nanopore sequencing is highly applicable to a variety of sequencing projects. In addition, we present the annotated reference genome sequence of ATCC 10970 with a detailed analysis of the biosynthetic gene clusters.

## INTRODUCTION

Streptomyces rimosus ATCC 10970 (NRRL 2234; WT5260) is a Gram-positive, aerobic, filamentous actinobacterium producing the first broad-spectrum antibiotic, oxytetracycline (OTC) (1). Stimulated by prolonged commercial interest in this product, which is also used as the intermediate for semisynthesis of the important extended-spectrum analogue doxycycline, numerous advanced genetics and molecular biology approaches have been developed (1). The first genome sequence of the *S. rimosus* ATCC 10970 strain was obtained in 2013 by Pethick et al. using a whole-genome shotgun sequencing approach and performed on a Roche 454 GS Junior apparatus (2). Soon after, the genome sequence of the industrial strain *S. rimosus* R6 (Pliva, Croatia) was also reported (3). To date, over 38 genome-sequencing projects of *S. rimosus* strains have been published in GenBank (4) (see Table S1 in the supplemental material). However, most of these genome sequences were of relatively low- quality, fragmented in several hundred contigs. Recently, we obtained a high-quality sequence of the *S. rimosus* ATCC 10970 strain using a combination of both long-read (PacBio) and short-read (Illumina) sequencing technology (GenBank assembly accession no. GCF_006229535.1). Using a similar approach, a group from the Korea Advanced Institute of Science and Technology (KAIST) (GCF_008704655.1) and a group from the University of Strathclyde (GCF_000331185.2) recently also obtained the genome sequence for this strain (5, 6).

*Streptomyces* species have complex genomes with high GC content often exceeding 70%. The genomes of these bacteria are primarily linear and can exceed 10 Mb in size. The chromosomes of actinobacteria such as *S. rimosus* often have a complex genetic organization with a highly syntenic central region flanked by variable chromosomal arms (7). *Streptomyces* species are known for their genome plasticity (7), including extensive DNA rearrangements, with repetitive DNA sequences often located in terminal parts of linear chromosomes containing simple telomeric DNA structures (8). These microorganisms can also contain one or more linear plasmids, sometimes reaching over 1 Mb in size and designated giant linear plasmids (GLPs) (9).

OTC-producing *S. rimosus* strains contain a single GLP of around 300 kb in size (1). The size of this plasmid varies between different strains of *S. rimosus*, and for many of them the GLPs have been detected and characterized. The industrial strain *S. rimosus* RCC 133 contains a linear plasmid, named pSRM, which is approximately 43 kb in size (10). A larger linear plasmid was detected in *S. rimosus* NCL with an estimated size of 255 kb (11). In two derivative strains of *S. rimosus* R6-501, namely, MBVI and MBV14, a GLP of 370 kb was found to be present (12). Pfizer’s *S. rimosus* strain also harbors the endogenous sex-factor plasmid SRP1 (13). A detailed analysis of the GLPs found in the Pliva pharmaceutical company’s *S. rimosus* strains was carried out (14, 15). *S. rimosus* R6-500 harbors a linear plasmid, pPZG101, 387 kb in size, known to have long (at least 95 kb) inverted repeats (15). Strain R6-65, which is the ancestor of the R6-500 strain, carries a smaller linear plasmid (pPZG102, 310 kb) and does not have long inverted repeats, leading to the conclusion that the long inverted repeats in pPZG101 occurred during strain development within the company (14). The analysis of 20 spontaneous morphological variants and 17 auxotrophic mutants of the *S. rimosus* R6 strain revealed strong plasmid-genome interactions and plasmid instability correlating with the mutant phenotypes. Two mutant strains completely lost the plasmid, and four independent mutants integrated parts of the plasmid within the chromosome (14). In one strain overproducing OTC, a large plasmid of approximately 1 Mb long (pPZG103) was identified. The increased size was the result of the integration of a chromosomal region with the OTC biosynthetic gene cluster (BGC) in the plasmid (14, 16).

Despite the rapid development of third-generation sequencing technologies (e.g., PacBio and Nanopore) the complex rearrangements of actinobacterial chromosomes and the potential interactions between chromosome and linear plasmid often make the whole-genome sequencing and assembly of these genomes rather challenging (17). While generating a high-quality genome sequence for the *S. rimosus* ATCC 10970 strain, we established an appropriate methodology for tackling *Streptomyces* genome complexity by applying both long-read and short-read sequencing technology, in combination with pulsed-field gel electrophoresis (PFGE). By applying PFGE and combining it with the bioinformatic pipeline for genome assembly, we validated our original *S. rimosus* ATCC 10970 genome sequence (genome sequence submitted under GenBank no. GCF_006229535.1) and published its revised and reannotated version with additional manual curation (https://morf-db.org/projects/TOPCAPI/streptomyces-rimosus). Additionally, PFGE was used for the specific isolation and extraction of the GLP DNA, followed by Nanopore sequencing. This way, we sequenced the linear plasmids separately from the chromosomal DNA for three different strains of *S. rimosus*—founder strain ATCC 10970 (GCF_006229535.1) and two industrial strains, M4018 (18) and R6-500 (3). We demonstrated that, despite having only a few nanograms of plasmid DNA isolated from the gel after PFGE separation, Nanopore technology represents a valid sequencing approach. This method ensured a clear separation of the plasmid DNA from the chromosome, thus further improving quality of both *S. rimosus* GLP and genome assemblies. By applying this methodology, we detected even small rearrangements occurring between the chromosome and the linear plasmid in the M4018 strain. Moreover, we also detected large and complex rearrangements and a large DNA duplication in the plasmid from the *S. rimosus* R6 strain. Our work points out that these complex rearrangements occurred due to numerous transposon mobilization events.

Finally, we also performed an analysis of the secondary metabolite biosynthetic pathways of strain *S. rimosus* ATCC 10970. The polished annotation of secondary metabolite gene clusters in conjunction with metabolite analysis also refined our knowledge of the secondary metabolite biosynthesis in these strains.

## RESULTS

### Streptomyces rimosus ATCC 10970 genome assembly and annotation.

PacBio sequencing of the genome of ATCC 10970 generated a total of 155,548 reads with an average GC content of 69% and a length ranging from 35 to 44,649 nucleotides with a substantial proportion of sequences in the 1,000 to 2,999 bp range (Fig. S1). Illumina sequencing of the ATCC 10970 strain genome generated a total of 186,867,024 short reads, 94.16% of which were of high quality (Q20) and 86.78% of which were of very high quality (Q30). When running multiple assembly pipelines, the best assembly result was obtained with the combination of Hybrid SPAdes and CANU, as shown in Table S2.

The genome sequence was assembled into a complete chromosome and a single plasmid, both having linear topology. Table 1 provides an overview of the assembly and annotation data. The GC content was slightly higher in the chromosome (at 72.0%) than in the plasmid (at 69.6%).

**TABLE 1:** Streptomyces rimosus WT genome assembly (GenBank accession no. GCF_006229535.1) and annotation data

Source	No. of bases	Circular	GC content (%)	No. of CDS	No. of tRNAs	No. of rRNAs
Chromosome	9,365,899	No	72.0	8,085	68	21
Plasmid	292,604	No	69.6	282		
Total	9,658,503		71.9	8,367	68	21

### Validation of the genome assembly of *S. rimosus* ATCC 10970.

Genome assembly was further validated by PFGE analysis. The BUSCO approach (19) was used to assess genome completeness and for the comparison with other publicly available genomic sequences of *S. rimosus*.

The *in silico* digestion of the total *S. rimosus* ATCC 10970 DNA with the rare-cutter restriction enzyme DraI resulted in 14 fragments. The majority of these fragments were visible on the PFGE gel, with the exception of three smaller fragments (<30 kb), which did not appear on the gel due to the PFGE running conditions (Fig. S2). Conversely, all 17 fragments predicted by the *in silico* analysis of the entire *S. rimosus* ATCC 10970 DNA based on the AseI enzyme digestion were detected on the PFGE gel, providing validation for the assembly (Fig. S2). These results agreed with PFGE analysis carried by Algora-Gallardo et al. (6).

Genome completeness was further validated using the BUSCO approach. Our assembly, together with the sequence in GenBank accession no. GCF_000331185.2, showed the highest level of completeness (99.7%) compared to all publicly available genome assemblies of different S. rimosus strains found in NCBI (Table S1). In total, 20 of the 35 available *S. rimosus* genomes showed genome completeness greater than 99%, but only four assemblies (GCA_006229535.1 [this study], GCF_000331185.2, GCA_008704655.1, and GCF_000707925.2) were marked with “complete genome” assembly status in the NCBI assembly database.

**FIG 1:**
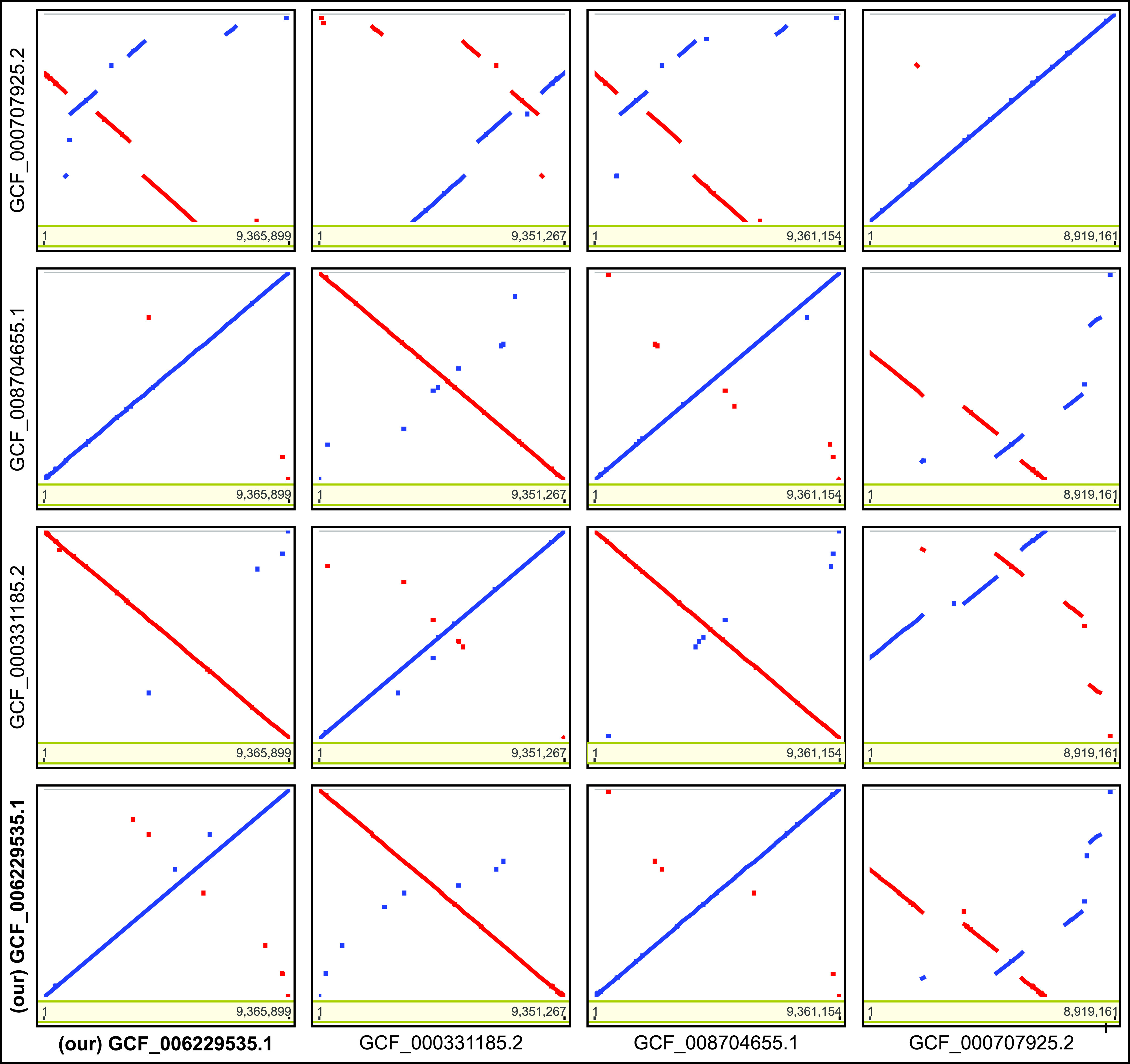
Dot plot alignment for four publicly available *S. rimosus* complete genome assemblies.

**FIG 2:**
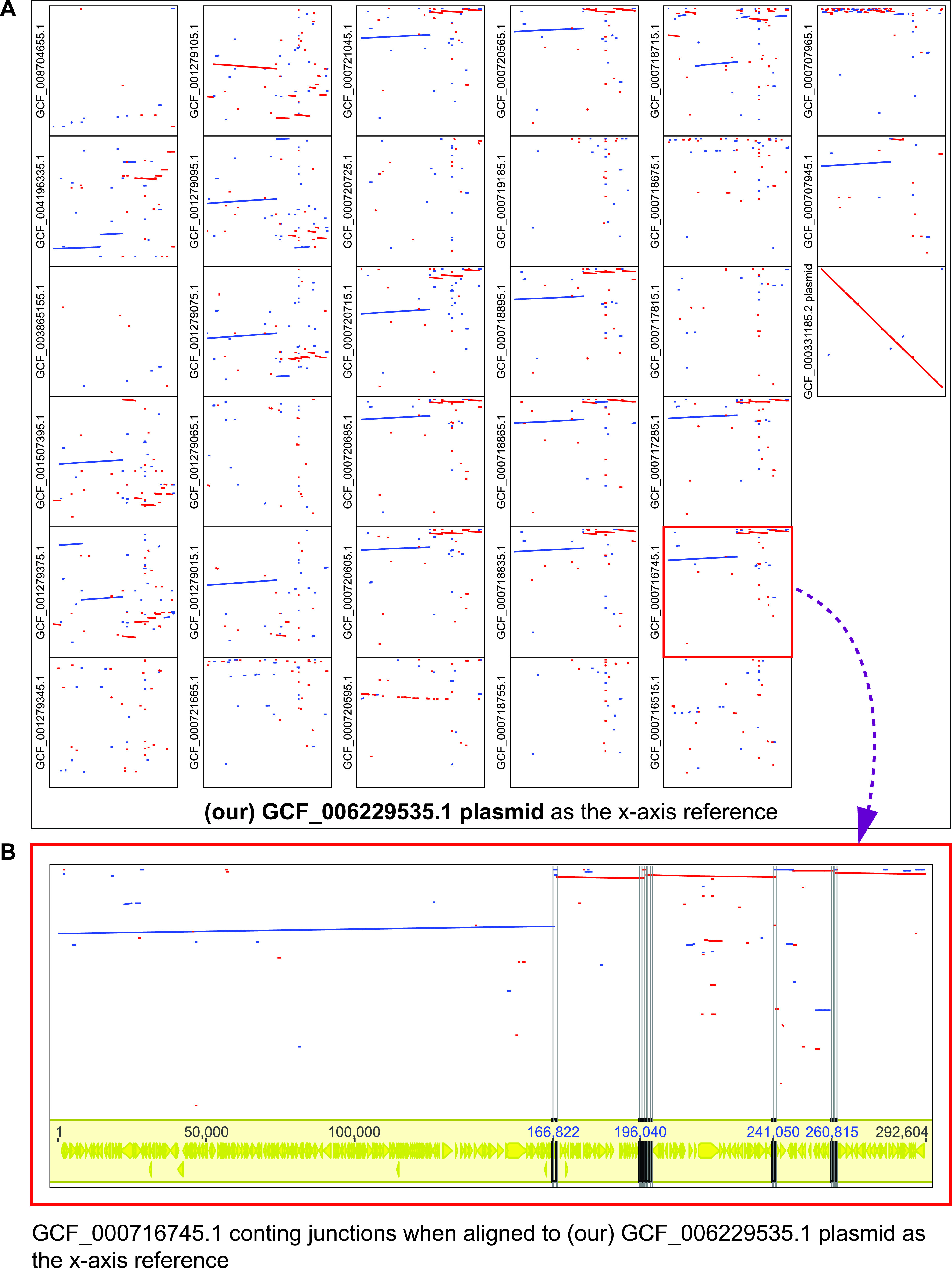
(A) Dot plot alignment between publicly available *S. rimosus* genome sequences and the GenBank GCF_006229535.1 plasmid assembly. (B) Genome assembly GCF_000716745.1 from strain NRRL B-8076 alignment to our GCF_006229535.1 plasmid assembly and highlighted junctions between aligned contigs. The highlighted junctions are coding sequences for transposases (Table S8).

The comparison with the publicly available complete genome assemblies (Fig. 1) revealed that the three assemblies of strain ATCC 10970 (NRRL 2234) could be aligned globally, across the entire chromosome length, whereas only those deposited under GenBank accession no. GCF_006229535 (this study) and GCF_000331185.2 had a complete plasmid sequence (Fig. 2). Among the three ATCC 10970 chromosome assemblies, ours is the largest (9,365,899 bp). When comparing it with the GCF_000331185.2 assembly, we identified the 14.6-kb difference between the two assemblies, including a 10-kb fragment (located at 4424878 to 4434878 bp) missing in the GCF_000331185.2 assembly, with 13 coding sequences encoding the GntR family transcriptional regulator, conjugal transfer protein TraS, mobile element transfer, replication initiation protein, and site-specific integrase. The sequence in assembly accession no. GCF_006229535.1 contains the 10-kb fragment and a reversed 6.7-kb section with TnsA-like and Mn transposases (located at 4680187 to 4686844 bp). GCF_008704655.1 appeared to lack the plasmid sequence completely. When comparing the assemblies of ATCC 10970 with the strain R6-500 (GCF_000707925.2), we observed numerous rearrangements and inverted regions in R6-500.

Compared to the GenBank accession no. GCF_000331185.2 genome assembly (6), our assembly lacked the complete telomere sequences at both ends of the chromosome (324 bp of missing sequence on the right and a 1,771-bp fragment on the left side). Regarding the linear plasmid telomeres, the plasmid from our ATCC 10970 assembly had the right arm containing a complete telomere sequence, while the left arm was missing a 20-bp fragment at the end, which was present on the plasmid sequence of GCF_000331185.2 (6).

### Giant linear plasmid (GLP) comparison of publicly available *S. rimosus* genomes.

Comparison of all publicly available genomic sequences with our plasmid assembly (Fig. 2A) revealed that only the aforementioned assembly of strain ATCC 10970 (GenBank accession no. GCF_000331185.2) (6) had a comparable plasmid sequence. Several contig-level assemblies had complete coverage of the plasmid sequence, but the contigs were not connected at certain regions. Interestingly, closer examination of these regions (Fig. 2B) revealed that all these regions connecting contigs contained different transposase genes (Table S8).

### PFGE separation and plasmid DNA extraction followed by Nanopore sequencing for plasmid assembly validation.

**FIG 3:**
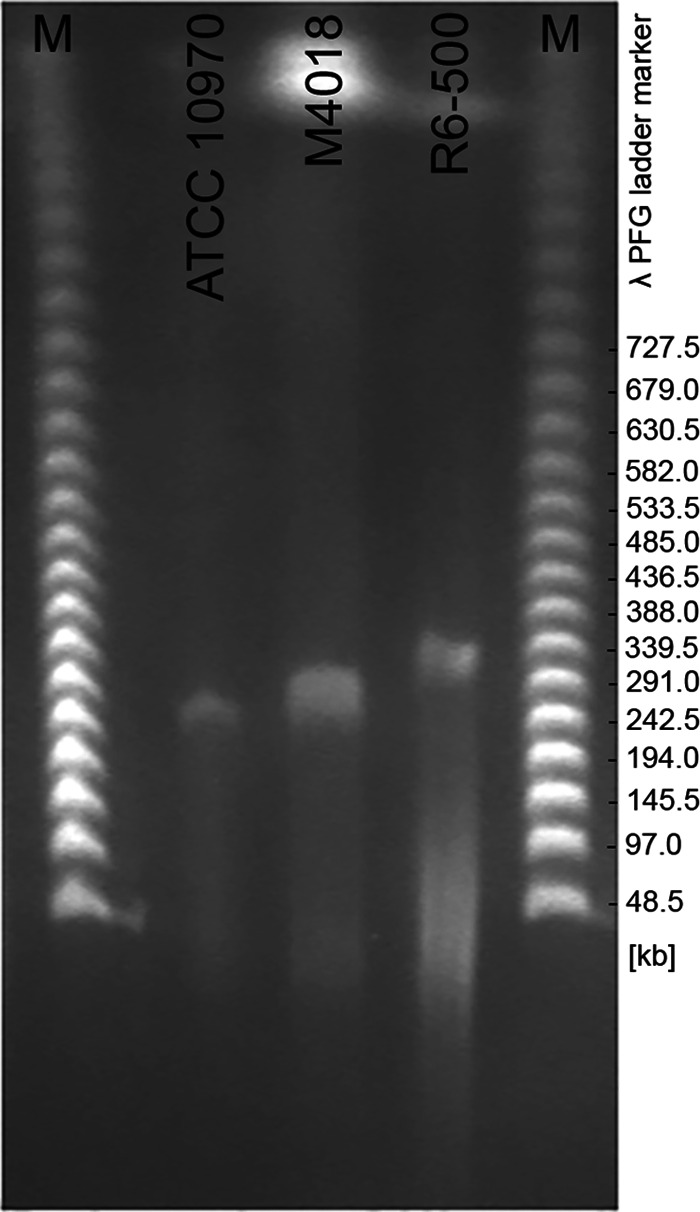
Plasmid DNA of *S. rimosus* ATCC 10970, M4018, and R6-500 separated by pulsed-field gel electrophoresis (PFGE). Determination of plasmid size in the *S*. *rimosus* strains ATCC 10970, M4018, and R6-500. M, lambda PFG ladder marker (New England BioLabs).

To validate the correct extraction, sequencing, and assembly of plasmid DNA from the overall DNA mixture, we developed a method for plasmid DNA extraction and direct sequencing using the Nanopore platform (see “Pulsed-Field Gel Electrophoresis for Plasmid Extraction” and “Oxford Nanopore Sequencing of Plasmids and *De Novo* Assembly”). By applying this PFGE method, total DNA was first embedded in low-melting-point agarose blocks, which were then loaded onto a PFGE gel for separation of plasmid DNA from the remaining chromosomal DNA. Using this approach, we separated the plasmid from the genomic DNA for the three strains considered in this study. Plasmids from *S*. *rimosus* ATCC 10970, M4018, and R6-500 were separated and visualized on the PFGE gel, confirming their presence in all three *S*. *rimosus* strains (Fig. 3). As visible on the PFGE gel, the plasmids of ATCC 10970 and M4018 appeared approximately the same size. However, the plasmid of R6-500 was larger (approximately 48 kb larger based on the comparison to a lambda PFG ladder marker). The plasmid bands were cut out from the PFGE gel and extracted as described in Materials and Methods. The extracted plasmid fragments were at a very low concentration (below 5 ng/μL) but still sufficient for the Nanopore sequencing.

Single-molecule Nanopore sequencing results and Canu assembly information are presented in Table 2. The Canu assembly pipeline successfully recovered three large contigs of 299,081 bp, 291,520 bp, and 189,364 bp, respectively, with coverage of 39.35×, 53.11×, and 86.45× for samples M4018, ATCC 10970, and R6-500, respectively. In addition, a small number of additional contigs (Table 2) were assembled, characterized by much lower coverage. BLASTn analysis suggested that these additional contigs are most likely debris from the *Streptomyces* chromosome, and they were therefore filtered out. The assembled sequence from the ATCC 10970 plasmid obtained from Nanopore sequencing was identical to the sequence obtained from PacBio and Illumina whole-genome sequencing, confirming that the plasmid hybrid assembly based on a combination of Illumina and PacBio reads was valid.

**TABLE 2:** Overview of Nanopore sequencing and Canu *de novo* assembly of isolated plasmid DNA

Features	M4018	ATCC 10970	R6-500
No. of base-called sequences	161,092	81,553	653,342
Yield of base-called sequences (Mb)	308.88	252.43	1733.66
Control lambda phage reads	45,408	6,400	82,806
Base-called sequences for assembly (Mb)	175.01	232.06	1472.47
Avg read length (bp)	1,512.86	3,087.81	2,580.85
Longest read length (bp)	25,954	39,848	253,123
Avg GC content (%)	66.79	68.03	68.63
Assembled contig (bp)	299,081	291,520	189,364
Coverage (×)	39.35	53.11	86.45
Additional contigs	7	14	3
Contig lengths (bp)	1,516–7,273	3,220–13,771	3,424–6,784

The GLP in *S. rimosus* R6-500 was originally characterized as pPZG101 (14). PFGE data (14) confirmed the presence of a slightly smaller linear plasmid in ATCC 10970 (R7) and M4018 compared to R6-500, which is in agreement with our PFGE results (Fig. 3). The estimated size of the ATCC 10970 and M4018 plasmids was 312 kb (14); the validated assembly here revealed slightly different sizes of 292.6 kb and 299.3 kb, respectively.

**FIG 4:**
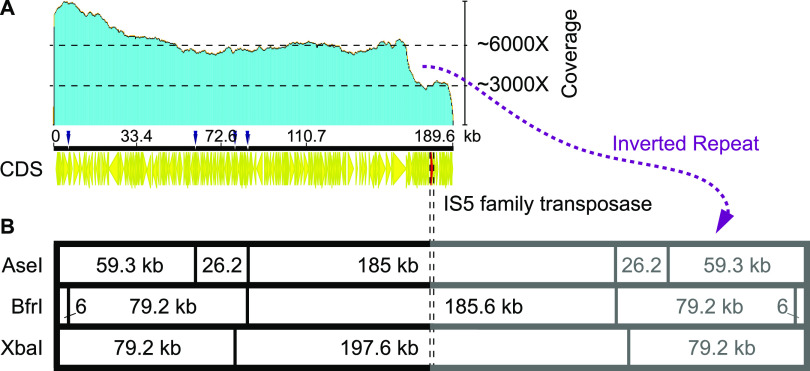
R6-500 plasmid analysis. (A) Coverage of Nanopore reads mapped to the R6-500 plasmid assembly. The proposed 167-kb inverted repeat has twice the coverage of Nanopore reads compared to the central region (167 to 190 kb); CDS, annotated coding sequences. The only unduplicated coding sequence in the central region, an IS*5* family transposase (R6500_083610), is highlighted in red at 173.6 kb. (B) *In silico* digestion of our R6-500 plasmid assembly with AseI, BfrI, and XbaI enzymes.

For *S*. *rimosus* R6-500, the plasmid size estimated by Gravius et al. is 387 kb (14). Based on our PFGE data, the estimated size of the R6-500 plasmid was expected to be smaller, ∼340 kb (Fig. 3). However, our Nanopore assembly yielded a significantly shorter plasmid contig (only 189.4 kb). When considering the coverage of mapped Nanopore reads to our assembly (Fig. 4A), the proposed long inverted repeat (167 kb) had twice the coverage of the proposed central region, further suggesting that the assembly was correct except that the long inverted duplications could not be separated by the assemblers. This suggests that the inverted repeat in our assembly was approximately 167 kb long, predicting the total size of the plasmid to be 356 kb, with the central region spanning over 20 kb. Interestingly, even the central region consisted largely of scattered duplications (Table S6), with the only coding sequence not being duplicated located in the central region—an IS*5* family transposase (R6500_083610), highlighted at 173.6 kb in Fig. 4.

### Comparison of large linear plasmids.

Some general features of the plasmids found in the three strains studied here (ATCC 10970, M4018, and R6-500) are summarized in Table 3. The linear plasmid found in the ATCC 10970 strain (*S. rimosus* ATCC 10970) was 292,604 bp long and contained 282 annotated coding sequences (CDSs). More than half of the predicted CDSs were annotated as hypothetical proteins.

**TABLE 3:** General features of the plasmids found in the three strains^*a*^

Strain	Length (bp)	GC content (%)	CDS (no.)	Median CDS length (bp)	Transposases (no.)	Transcriptional regulators (no.)	Hypothetical proteins (no.)
WT5260	292,604	69.6	282	621	21	18	148
M4018	299,299	69.5	291	618	23	18	152
R6	189,563 (∼356 kbp)	69.8	202 (386)	564	14 (24)	11 (21)	117 (225)

a The numbers in parentheses are the number of features with the proposed large inverted repeat in strain R6.

To further elucidate the functional relevance of the plasmid-encoded parts of the genomes, we compared the complement of enzymes encoded on the ATCC 10970 plasmid to an unpublished genome-scale metabolic model of *S. rimosus*. We found that none of the plasmid genes code for predicted metabolism-related enzymes, thus suggesting that GLPs do not harbor essential gene homologues.

**FIG 5:**
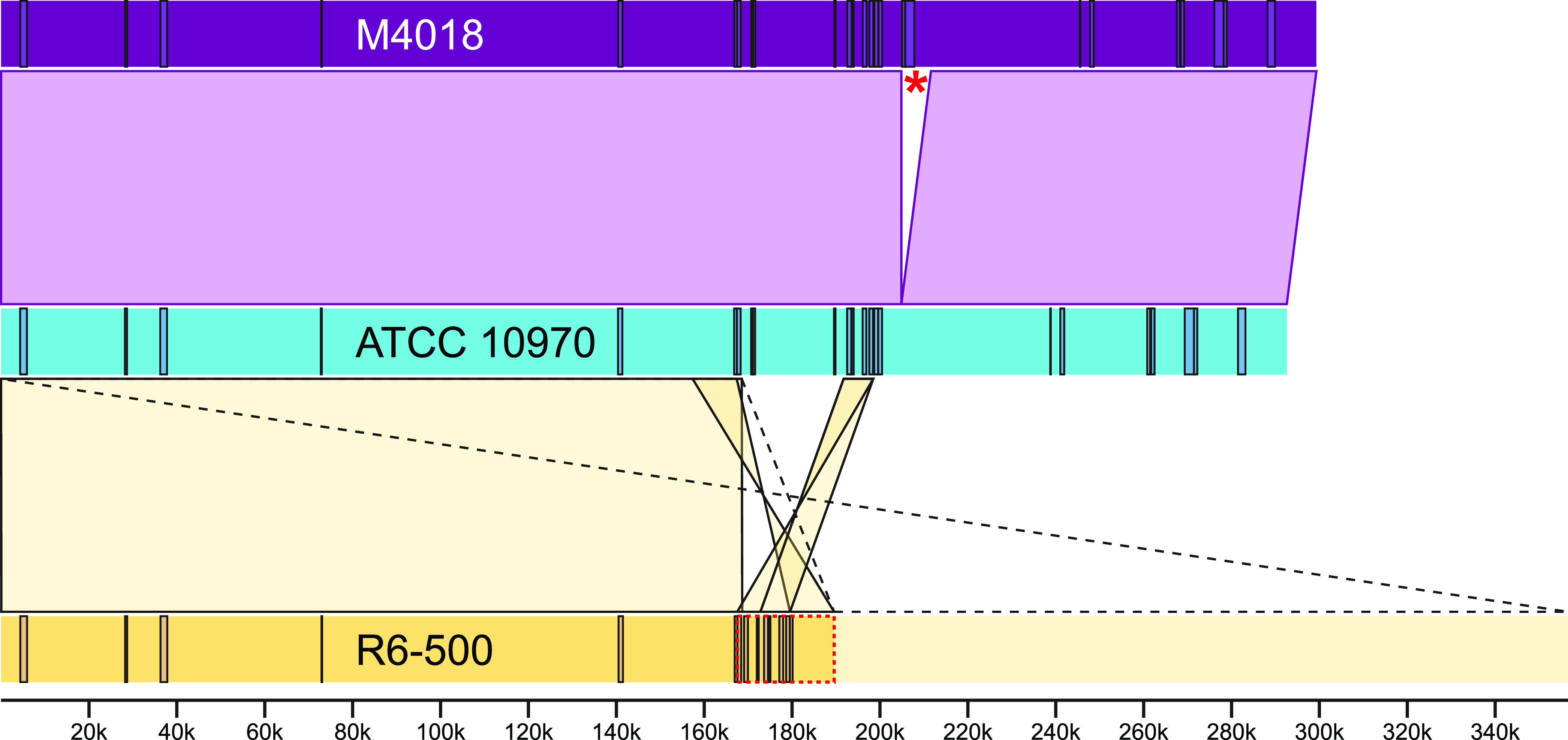
Schematic representation of the rearrangements of the DNA sequences of the linear plasmids from ATCC 10970, M4018, and R6-500. Rectangles, annotated transposase-related coding sequences for each plasmid sequence; Asterisk, 6.7-kb transposase fragment from the chromosome present in the M4018 plasmid; ribbons, ATCC 10970 plasmid rearrangements in M4018 and R6-500 plasmid; dotted ribbon, proposed long inverted repeat in the R6-500 plasmid; red dotted rectangle, central region of the R6-500 plasmid, consisting largely of scattered duplications; scale, size of the genomic sequences in bp.

Since ATCC 10970 is the parental strain of both industrial strains M4018 and R6-500, we compared the rearrangements in their plasmids to the parent plasmid (Fig. 5). For the R6-500 (pPZG101) plasmid, the left arm of the plasmid was confirmed to be similar to the ATCC 10970 plasmid, whereas the right arm of the plasmid was different (14). The conserved left arm of the R6-500 and ATCC 10970 plasmids terminated in a region characterized by a high frequency of transposase genes; 11 of a total of 23 plasmid transposase genes were found within this 40- kb region (from 160 to 200 kb on the ATCC 10970 plasmid). Further rearrangements in the central region of the R6-500 plasmid (167 to 188 kb on the R6-500 plasmid) were also consistent with the presence of these transposase genes. Transposase-coding sequences were also observed when comparing the ATCC 10970 and M4018 plasmids, with the M4018 plasmid containing a novel 6.7- kbp transposase region translocated from the chromosome (Fig. 5).

The plasmid found in the M4018 strain was slightly larger than the one found in the ATCC 10970 strain, with all of the CDSs found in the ATCC 10970 plasmid being conserved. The increased size of the M4018 plasmid was due to an additional 6,708-bp fragment originating from the chromosome, which was transferred to the plasmid and inserted in position 204,853 (Fig. 5). This DNA fragment was found to contain 9 CDSs, which are summarized in Table S3; among them, we also observed a Mu transposase domain. In the ATCC 10970 chromosome, this region was found to exist in two copies, first at 4680190 to 4686883 and the second at 6119904 to 6113211 bp.

Both the M4018 and R6-500 plasmids were aligned with the ATCC 10970 plasmid using progressiveMauve (20), which allowed the identification of several single nucleotide polymorphisms (SNPs) in the M4018 and R6-500 plasmids, which are listed in Table S4 and Table S5, respectively. In M4018, six of these resulted in a predicted change in the encoded protein sequence, while two resulted in a disruption of the reading frame. As for R6-500, alignment revealed that the majority of the R6-500 plasmid sequence, extending from position 1 to position 168654, was nearly identical to the first half of the ATCC 10970 plasmid. The only differences were represented by 14 mutations, none of which resulted in a predicted change in the encoded protein sequence, while 9 resulted in a disruption of the reading frame. In the 20,910-bp-long central region of the R6 plasmid, we observed rearrangements and inversions compared to the ATCC 10970 plasmid (Fig. 5). The 24 CDSs present in this part of the plasmid are summarized in Table S6. Interestingly, they include seven transposase genes, two of which are duplicates.

### Biosynthetic gene clusters encoding secondary metabolite biosynthesis in the *S. rimosus* genome.

**FIG 6:**
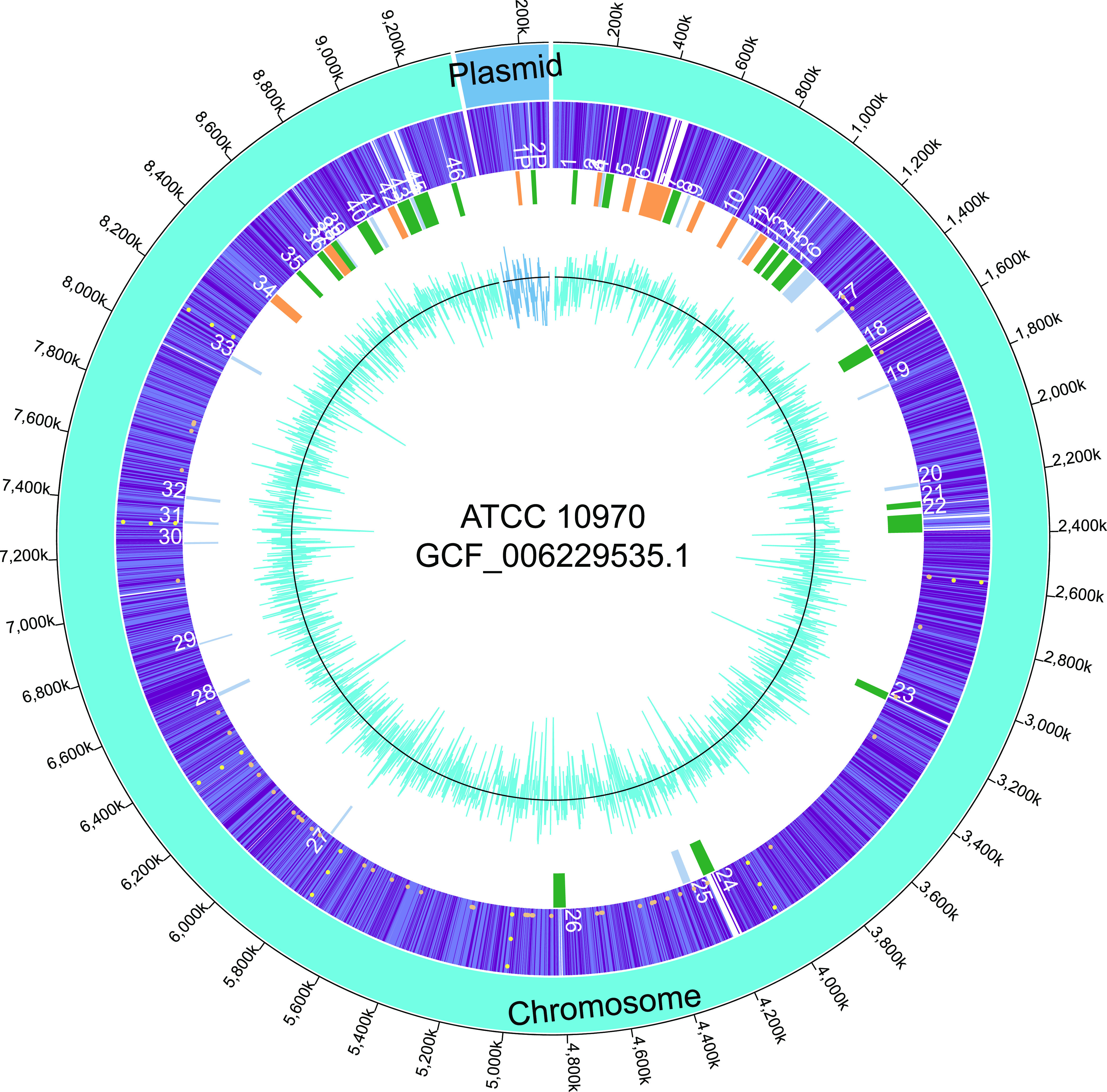
Schematic representation of the whole genome for strain ATCC 10970 (GenBank assembly no. GCF_006229535.1). First/outer ring, size of the genomic sequences in bp; second ring. Plasmid and chromosome identification. Third ring, forward/reverse CDSs; marked dots show functional RNA elements (orange, tRNA; yellow, rRNA); numbered BGCs. Forth ring, marked BGCs; orange indicates polyketide synthase-like clusters, green indicates nonribosomal peptide-like clusters, and blue indicates remaining clusters. Fifth ring, GC content (colored line) and GC average at 71.9% (black line).

For the identification of putative biosynthetic gene clusters (BGCs) in the PFGE validated genome assembly of *S. rimosus* ATCC 10970 and in the plasmid sequences of ATCC 10970, R6-500, and M4018, we used antiSMASH 6.0 (21) and manually curated the acquired data. In the chromosome of the ATCC 10970 strain, we identified 46 putative BGCs, and only 2 BGCs were located on the plasmid (Fig. 6, Table 4. The plasmid from the M4018 strain also contained two putative BGCs; however, the R6-500 strain plasmid only harbored one. The distribution of the putative clusters on the chromosome of ATCC 10970 strain was nonrandom, marked by BGC abundance on both terminal arms, which is characteristic for actinomycetes. Identified putative gene clusters represented around 14% of the whole genome in *S. rimosus* ATCC 10970.

**TABLE 4:** Putative biosynthetic gene clusters in the *S*. *rimosu*s ATCC 10979 genome and isolated metabolites in our study

Cluster no. in ATCC 10970	Type	Position from	Position to	Most similar known biosynthetic gene cluster (percent similarity)	Metabolites detected in extract in our study
Chromosome					
1	NRPS fragment	90930	97183	Paromomycin (7)	Guanipiperazines A and B
2	PKS type I-NRPS	188819	209096	NA	
3	Terpene	209047	217564	Isorenieratene (85)	
4	NRPS	225846	253508	Atratumycin (13)	
5	Type I PKS	321687	347936	Sceliphrolactam (32)	
6	Type I PKS	399364	499930	Nystatin A1 (72)	Rimocidin, CE108, amide CE108
7	NRPS	513458	544839	Qinichelins (22)	
8	Lassopeptide	579166	586929	Lagmysin (80)	
9	Type II PKS	628015	655782	Oxytetracycline (100)	Oxytetracycline
10	Type I PKS	786388	806568	NA	
11	Lantipeptide	899955	907971	NA	
12	Type I PKS	922668	952762	Spiroindimicins/indimicins/lynamicins (6)	
13	NRPS like	989591	1015728	Stenothricin (13)	
14	NRPS-PKS type	1034416	1064312	Rimosamide (92)	Rimosamides A–D
15	NRPS	1095198	1140552	Daptomycin (14)	
16	Arylpolyene	1162316	1218483	Herboxidiene (3)	
17	Terpene	1386125	1399202	Hopene (76)	
18	NRPS	1568818	1619165	Isocomplestatin (93)	
19	Melanin	1756702	1763509	Bagremycin A/B (11)	
20	Lantipeptide	2189994	2200974	NA	
21	NRPS	2267432	2288427	Streptobactin (70)	
22	NRPS	2320795	2393710	Ulleungmycin (36)	
23	NRPS-PKS type	3089234	3116494	Tyrobetaine (100)	Tyrobetaine, tyrobetaine-2, chlorotyrobetaine, chlorotyrobetaine-2
24	NRPS	4147387	4194710	Mannopeptimycin (22)	
25	Arylpolyene	4258214	4287270	Fusaricidin B (25)	
26	NRPS	4793268	4840550	Ishigamide (61)	
27	Lassopeptide	5834963	5841023	Moomysin (50)	
28	Lantipeptide	6587454	6598475	SAL-2242 (77)	
29	Terpene	6817266	6819473	Geosmin (100)	
30	Ectoine	7244554	7247941	Ectoine (100)	Ectoine, hydroxyectoine
31	Siderophore	7331013	7336394	Desferrioxamine E (100)	
32	Siderophore	7433301	7442083	NA	
33	Terpene	8052420	8062100	NA	
34	Type I PKS-NRPS	8343488	8380063	Marinacarboline (23)	
35	NRPS	8502626	8519135	Deimino-antipain (66)	Chymostatin A, B, C
36	NRPS like	8619558	8643234	NA	
37	PKS type I or PKS type I-saccharide	8655191	8687260	Tetronasin (9)	
38	NRPS	8692521	8715452	Mannopeptimycin (14)	
39	Terpene	8720327	8725815	NA	
40	Other-NRPS like	8825293	8867032	A83543A (8)	
41	Butyrolactone	8884982	8896849	Cyphomycin (11)	
42	PKS type I-NRPS	8971199	8996615	NA	
43	NRPS	9016185	9065343	Teicoplanin (28)	
44	Nucleoside	9075785	9088816	Pseudouridimycin (68)	Pseudouridimycin
45	NRPS	9091105	9149322	NA	
46	NRPS	9257979	9275999	NA	
Plasmids					
1P	Type I PKS	143989	163050	Kanamycin (1)	
2P	NRPS	215829	230795	NA	

We can divide the BGCs encoded by *S. rimosus* ATCC 10970 into three categories: (i) BGCs whose products have been identified, (ii) BGCs with very high similarity to known BGCs whose products have been identified in other actinomycetes, and (iii) predicted BGCs with little or no homology to any known BGCs. We collected all the information on secondary metabolites produced by *S. rimosus* species available in literature. Here, we also present the results of our own analysis of secondary metabolites in strain ATCC 10970 using liquid chromatography-mass spectrometry (LC-MS) and LC-UV-high-resolution mass spectrometry (HRMS).

In our data mining analysis (Table 4), we identified oxytetracycline (BGC no. 9, Table 4) as polyketide synthase (PKS) type II BGC. In addition to OTC, we identified a known metabolite, rimocidin, a PKS type I metabolite designated BGC no. 6, which was recognized with antiSMASH as nystatin A1 BGC (Table 4). Other known metabolites, such as rimosamide (BGC no. 14) and tyrobetaine (BGC no. 23), were also found. All 48 putative metabolites (2), such as desferrioxamine (BGC no. 31), were also found in our analysis. We identified two additional ones with 100% similarity to known clusters producing previously characterized metabolites, the volatile compound geosmin (BGC no. 29) (22, 23) and the osmolyte ectoine (BGC no. 30) (24, 25).

**FIG 7:**
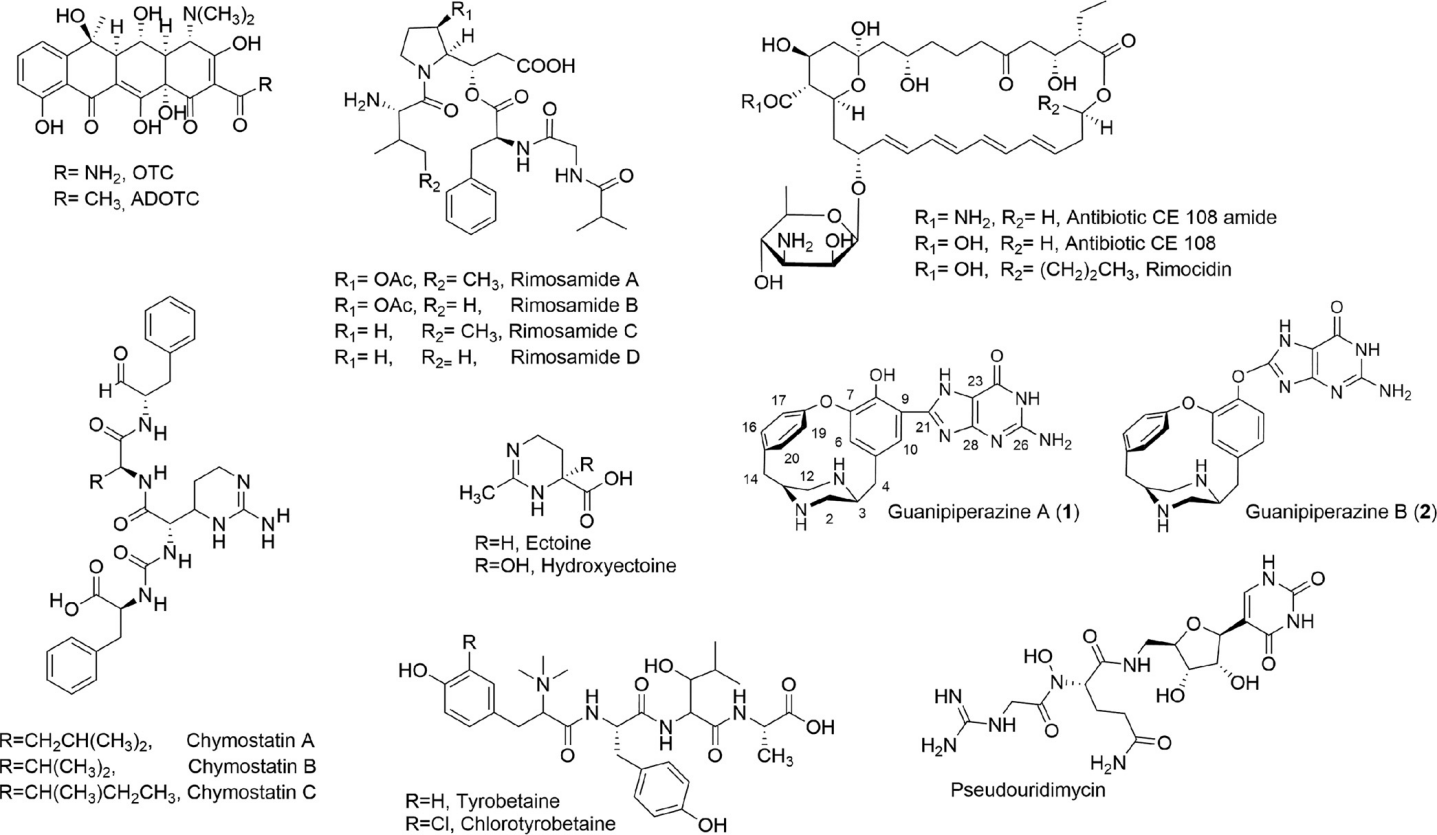
Structures of secondary metabolites detected in the extract from *S. rimosus* ATCC 10970.

Since ATCC 10970 is the parental strain of the industrial strains, we analyzed extracts from the culture of ATCC 10970 by LC-MS and LC-UV-HRMS to identify the secondary metabolites present and correlate this information with the biosynthetic gene clusters encoding the secondary metabolites (Table 4). As expected, the chromatographic profile of the acetonitrile extract revealed the presence of oxytetracycline, the antibiotic rimocidin, and CE108 as major components of the extract (Fig. S3). As minor components of the acetone extract, we detected rimosamides A to D (BGC no. 14), which were previously described as metabolites of *S. rimosus* (26). In the pellet extracts using the solvents methanol:acetone we detected the osmolytes ectoine and hydroxyectoine (encoded by BGC no. 30) (Table S10), the nonribosomal peptide-synthetase (NRPS)-encoded metabolites chyhmostatins A, B, and C (BGC no. 18) (Table S10) and tyrobetaines (tyrobetaine, chlorotyrobetaine, dichlorotyrobetaine, tyrobetaine-2) (BGC no. 23) (Fig. 7).

In the genome of *S*. *rimosus* ATCC 10970, we identified one nucleoside BGC (Table 4; BGC no. 44) with 68% similarity to the pseudouridimycin (PUM) cluster (BGC0001476) from *Streptomyces* sp. strain ID38640 (27). The comparison of the *S*. *rimosus* BGC with the pseudouridimycin BGC from Streptomyces sp. ID38640 revealed high protein similarity (Table S13). Compared with the known pseudouridimycin cluster, BGC no. 44 does not include *pumA*, *pumB*, *pumC*, or *pumD* homologue genes. The function of PumA is unknown in the biosynthesis of PUM. PumB is a putative exporter, and PumC is a predicted DeoR transcriptional regulator. PumD is a putative hydrolase of the HAD family with the predicted function in the initial steps of biosynthesis (27, 28). Despite the absence of *pumA*, *pumB*, *pumC*, and *pumD* homolog genes in BGC no. 44, we detected production of PUM in our S. rimosus ATCC 10970 (Table S10).

Guanipiperazines A and B were isolated after extract fractionation by SP207SS column chromatography and preparative reversed-phase high-pressure LC (HPLC) (supplemental material- extraction and isolation guanipiperazines A and B), and their structures were characterized by one-dimensional (1D) and (2D) nuclear magnetic resonance (NMR) data analysis (Table S11). This confirmed the expression of the *rms* BGC in the ATCC 10970 strain, which was recently characterized in the heterologous host Streptomyces lividans TK24 (29). To identify the BGC encoding these compounds, we performed a BLAST analysis using the noncanonical NRPS LnaA, which is involved in piperazine biosynthesis (29), as a query. We found a region of 6,254 bp (positions 90930 to 97183 of the genome), designated *rms*, that is nearly identical to the BGC described for guanipiperazine (*gup*) (30) and is characterized as BGC no. 1, which was not recognized by the antiSMASH analysis. The *rms* BGC encodes a noncanonical NRPS, two P450 cytochromes, and an F_420_H_2_-dependent reductase structurally related to pyridoxamine 5′-phosphate oxidases (Table S12).

A number of GLPs from *Streptomyces* have been characterized, and some of them encode clusters for biosynthesis of antibiotics (31). The giant linear plasmid of *S*. *rimosus* ATCC 10970 and M4018 harbors two putative BGCs with identical nucleotide sequences (Table S7). On the other hand, R6-500 GLP only harbors one BGC in two copies due to the long inverted repeat. BGC no. 1P was present on GLP in all three strains, wild type (WT), M4018, and R6-500. This is a PKS type I cluster, with one major PKS gene (WT5260_008341) containing domains organized in the following manner: KS-AT-DH-ER-KR-PCP, where based on the *in silico* prediction, the AT domain incorporates a malonyl-CoA or methylmalonyl-CoA unit. Beside the PKS gene in BGC no. 1P, there were five additional hypothetical proteins identified, two transcriptional regulators and a cytochrome P450 protein. Further analysis of the PKS type I gene (WT5260_008341) from BGC no. 1P using MIBiG hits from antiSMASH predicted closest homologues in NRPS-PKS gene clusters of thaxteramide A1 (BGC0002026), chondramide A (BGC0000969), or epothilone (BGC0000991), where homologs of PKS gene WT5260_008341 form part of the PKS-NRPS enzyme complex. In contrast, most BLAST hits related to this PKS gene (WT5260_008341) revealed homology to mycocerosic acid synthase (MAS)-like PKS enzyme from *Myxobacteria* involved in the iterative biosynthesis of long- and branched-chain fatty acids (32). Clusters containing standalone PKS modules were recently characterized in an iterative assembly of linear intermediates in *Streptomyces* species (33); therefore, we predicted BGC no. 1P found on *S*. *rimosus* GLP to operate iteratively, considering that this cluster also consisted of a standalone PKS gene (WT5260_008341) and small number of auxiliary genes.

The other identified BGC, no. 2P, located on GLP of WT and M4018 belongs to the NRPS class, with no close similarity to previously studied BGCs. This NRPS BGC was found to contain C-A-PCP-C-A-PCP-TE domains, and based on the *in silico* prediction, it should incorporate Ser and Pro amino acids. Three genes from BGC no. 2P were located on the plasmid (NRPS, WT5260_008400; FAD-binding protein, WT5260_008401; oxidoreductase, WT5260_008402) and displayed 66 to 69% nucleotide identity with cluster 46, present on the right end of the linear chromosome. Many other unknown metabolites are likely encoded in the *S. rimosus* ATCC 10970 genome (Table 4), and their detailed analysis may reveal hidden potential for new natural compounds. The structures of identified secondary metabolites are presented in Fig. 7.

## DISCUSSION

The whole-genome assembly of ATCC 10970 was validated by its PFGE digestion profile and compared with two previously published PFGE results (34, 35). The restriction of the chromosomal DNA obtained from the ATCC 10970 (R7) strain, performed by Pandza et al., yielded 14 visible fragments after AseI digestion. This agreed with our data, since three additional fragments in our PFGE gel originated from the plasmid DNA. The only difference was in the estimated fragment sizes; the published lengths of Pandza et al. differed for the larger bands (>500 kb), which is likely to be a consequence of the high GC content and more complex estimation of fragment length when using DNA size markers (35). Our PFGE analysis was also consistent with recently published PFGE data (6). We obtained the same number of fragments after *in silico* restriction, and the visible DNA fragments on the PFGE gel correlated strongly. However, there was a slight difference in the length of the *in silico* fragments compared to our data due to a missing 10-kb fragment in the GenBank GCF_000331185.2 assembly (6).

Using a combination of Hybrid SPAdes and Canu assemblers, we achieved the highest ND50 value and the largest contig size reported. It is worth mentioning that the reads displayed marked redundancy and duplications of some k-mers, which can be explained by the presence of several long repeats in *Streptomyces* sp. genomes (36). The final genome sequence was assembled into a complete chromosome and a single plasmid, both with linear topology.

The genome completeness of our assembly (99.7%) was matched by two other publicly available assemblies (GenBank accession no. GCF_000331185.2 and GCF_000717285.1), with marginal differences in the numbers of duplicated, fragmented, and missing BUSCOs. All three assemblies originating from strain ATCC 10970 showed a high degree of similarity. This implies that the conservation level of genes under the BUSCO order *Streptomycetales* for *S. rimosus* species could be as high as 99.7%, which should serve as the reference for future *S. rimosus* assembly quality assessments. The high degree of similarity and further validation of the correctness of the assembly was confirmed by comparison with publicly available complete genome assemblies. All three chromosome-level assemblies of ATCC 10970 could be aligned globally. In the GCA_008704655.1 assembly, the plasmid sequence was completely absent. Among the three ATCC 10970 chromosome assemblies, ours was the largest (9,365,899 bp), but it still lacked complete telomere ends in comparison to GCF_000331185.2 (6).

By applying antiSMASH, we identified 48 BGCs. As usual with *Streptomyces* genomes, most BGCs were located toward the extremities of the linear chromosome. In the parental strain, two gene clusters were located on the GLP, while in strain R6-500, one BGC was apparently lost when part of the plasmid was deleted after duplication. Compared to previous studies (2) we identified two additional putative BGCs with 100% similarity to known clusters producing previously characterized metabolites, the volatile compound geosmin (22, 23) and the osmolyte ectoine (24, 25). Geosmin has been isolated from several *Streptomyces* species, and its production in *S. rimosus* can be predicted based on high similarity with published BGC sequences. Two identified BGCs of *S. rimosus* ATCC 10970 displayed high similarity with already published clusters, and therefore we could hypothesize that similar metabolites can be produced by the ATCC 10970 strain—carotenoid isorenieratene (BGC no. 3) (85% similarity) (37, 38) and isocomplestatin (BGC no. 18) (93% similarity) (39).

From previous studies, *S. rimosus* ATCC 10970 was known to produce oxytetracycline, rimocidin (1), rimosamide (26), and tyrobetaine (40), and we confirmed their biosynthesis in our experiment. Interestingly, pseudouridimycin was also detected in our extracts, although 4 *pum* genes from the homologous *pum* BGC (28) were absent. We also detected the rimocidin analogs CE108 and amide CE108, previously characterized only in strain Streptomyces diastaticus var. 108 (41, 42). For the first time, we confirmed in *S. rimosus* ATCC 10970 the production of the common osmolytes ectoine and hydroxyectoine, produced by many *Streptomyces* species as a response to hyperosmotic and heat stress (43). Recently Maxson et al. identified chymostatin A, B, and C in *S. rimosus* subsp. *rimosus* NRRL B-8076 (44). We identified the same NRPS metabolite in *S. rimosus* ATCC 10970. Although guanipiperazines A and B were recently isolated by heterologous expression in S. lividans TK (29), this is the first time these compounds were detected and isolated from an extract of a native producer such as *S. rimosus* ATCC 10970. We identified the BGC responsible for the biosynthesis of guanipiperazines A and B in the genome of *S. rimosus* ATCC 10970, and while the cluster appears to be cryptic in many other *Streptomyces* species (30), the isolation and identification of guanipiperazines confirmed that the biosynthetic pathway is expressed in this strain. Despite the many bioinformatic tools available, such as antiSMASH, detailed manual analysis is necessary to gain a deeper understanding of the BGCs encoded and their products or to predict metabolites not yet identified.

The comparison of the GLP assembly with all publicly available genomic sequences of *S. rimosus* revealed that only the assembly of GenBank accession no. GCF_000331185.2 (6) had a complete plasmid sequence, with our assembly missing a 22-bp telomere sequence at the left end of the plasmid. Therefore, our Nanopore sequencing approach combined with PFGE extraction of the extrachromosomal element did not provide a complete telomere sequence. However, it did validate the correctness of the assembly. A total of 19 out of 33 *S. rimosus* assemblies had a similar distribution of aligned contigs as our ATCC 10970 plasmid sequence. A closer inspection revealed that several transposase genes were located at the ends of these contigs, suggesting that transposases play an important role in genome rearrangements. The role of transposases in the dynamics and rearrangements of *S. rimosus* sequences, especially in the plasmid sequence, became clear when the M4018 and R6-500 plasmids were sequenced and compared with the parent strain *S. rimosus* ATCC 10970. The M4018 plasmid harbored a 6.7-kb insertion with the transposase domain originating from the chromosome, and the R6-500 plasmid had a long (167-kb) inverted repeat with seven transposase genes located in the central region of the GLP, where rearrangements occurred and the inverted repeat began. The frequency of occurrence of transposase genes was significantly (approximately 10-fold) higher on the plasmid sequences (approximately 78.9, 83.6, and 59.0 transposase genes per Mbp for ATCC 10970, M4018, and R6-500 plasmids, respectively) compared to the chromosome having 5.7 transposase genes per Mbp. It is important to note that we did not detect essential genes, such as genes involved in primary metabolism, in the GLPs. Interestingly, despite intensive strain selection of the industrial strains R6-500 and M4018, GLPs appear to replicate stably over the years despite different types of selective pressure.

When comparing the GLPs found in three *S. rimosus* strains, we identified small differences between the GLP of M4018 and ATCC 10970 strains, including small rearrangements, several SNPs, and a larger chromosomal DNA integration in M4018 GLP. However, we also observed larger plasmid rearrangements in R6-500, such as inverted duplications, which were more difficult to address. DNA rearrangements are not uncommon in *Streptomyces* species (45, 46). Interaction or recombination between the *Streptomyces* chromosome and its plasmid is also a common event. For example, in Streptomyces coelicolor the SCP1 plasmid was integrated in the chromosome (47), and in S. lividans recombination between the SPL2 plasmid and the chromosome occurred (48). Gravius et al. also showed for *S*. *rimosus* R6-500 that recombination can occur between linear ends of GLP and chromosome (14). We have shown that *S. rimosus* GLPs can undergo very drastic rearrangements, and yet they seem to be stably maintained. It is therefore reasonable to assume that GLP in *S. rimosus* is somehow important for functionality of the entire cell. However, Gravius and collaborators have succeeded in isolating GLP-free mutants (14), showing that the GLP gene products are not essential for cell survival, at least not under laboratory conditions.

The correct assembly of the plasmid DNA sequence is particularly difficult due to small duplications and inversions. It seems that there is a shortage of assemblers that can handle large duplications. Therefore, to ensure the highest-quality sequencing and assembly of GLP in the three *S. rimosus* strains, we combined the PFGE extraction and Nanopore sequencing approach. Surprisingly, despite the very low concentrations of GLP DNA obtained from the gel after PFGE separation (in the range of a few nanograms), we were able to obtain high-quality DNA reads, clearly separating the GLP DNA from the rest of the chromosome. In this way, we were even able to identify the insertion of 6.7 kb from the chromosome and GLP found in strain M4018. Interestingly, the small 6.7-kb chromosomal fragment identified in the GLP of M4018 is flanked by a Mu-like transposase domain gene, again indicating a central role of transposons/insertion (IS) elements in the genome evolution of this *S. rimosus* strain.

We have demonstrated that it is possible to obtain a high-quality sequence of extrachromosomal DNA using this approach, even for DNA with high GC content. This approach could be utilized to ensure high-quality sequencing of each specific DNA fragment of chromosomal DNA after digestion by rare enzymes, demonstrating the usefulness of Nanopore sequencing in combination with PFGE. As previously observed by Gravius et al. (14), GLP DNA rearrangements in strain R6-500 were extensive. They constructed a restriction map and suggested that the ends of plasmid pPZG101 (from R6-500) were characterized by long inverted repeats of at least 95 kb. Pandza et al. later constructed an ordered a cosmid library from PFGE-isolated plasmid DNA of pPZG101. By applying restriction analysis and in combination with Southern hybridization, they were able to assemble an ordered cosmid library of the entire plasmid, demonstrating that pPZG101 contains long inverted repeats of approximately 180 kb and a unique central region of approximately 30 to 32 kb (15), as also shown schematically in the Fig. 4 and 5. *In silico* digestion (Fig. 4B) using the same restriction enzymes as Pandza et al. (15) revealed a very similar distribution of fragments for our R6-500 plasmid assembly. Using the approach combining PFGE and Nanopore sequencing and based on the work by Pandza et al. (15), we can conclude that a very large portion of the plasmid (one complete side) was deleted while duplicating the other plasmid end, as indicated by the doubling of coverage compared to the central region of the plasmid.

We failed in assembling the entire plasmid in R6-500. The difficulty in handling large inverted repeats appears to be the problem with both assemblers (Canu, Flye), and we were unable to separate the repeated ends. Interestingly, the very extensively rearranged short inner portion of the plasmid of at least 20 kb in strain R6-500 contains seven very densely scattered copies of different transposase gene homologs. This is quite extraordinary considering that we can identify only 53 transposase gene homologs in the entire 9.37-Mbp linear chromosome of this strain. Interestingly, these rearrangements resulted in large regions of perfect homology that appear to be stably conserved in the R6-500 strain. Sequencing of the GLP in three *S. rimosus* strains highlighted several rearrangements accompanied by transposase genes, suggesting that transposases play an important role in *S. rimosus* plasmid and genome plasticity.

## MATERIALS AND METHODS

### Strains.

Three Streptomyces rimosus strains were used in this study: (i) wild-type *S. rimosus* ATCC 10970 (NRRL 2234; WT5260), sometimes referred to as strain R7 (49), (ii) *S. rimosus* M4018 (DSM 105900), a Pfizer strain (50), and (iii) *S. rimosus* R6-500, derivative of Pliva strain R6-65 (51). To obtain spores, *S. rimosus* strains were plated on soya-mannitol agar (MS) (52) and incubated at 28°C for 7 days.

### Genomic DNA (gDNA) extraction.

For gDNA extraction of *S. rimosus* ATCC 10970, a plug of sporulating colonies on MS medium was inoculated into 5 mL tryptone soy broth medium (TSB; 17 g L^−1^ casein peptone, 3 g L^−1^ soy peptone, 2.5 g L^−1^ glucose, 5 g L^−1^ sodium chloride, and 2.5 g L^−1^ dipotassium hydrogen phosphate, pH 7.3) (52) and incubated at 28°C in a shaker at 220 rpm and harvested during the mid-exponential-growth phase (18– to 22 h of incubation). Extraction was performed using the GeneElute bacterial genomic DNA kit (Sigma-Aldrich, USA) according to the manufacturer’s instructions. The quality and quantity of extracted gDNA was assessed by agarose gel electrophoresis.

### Genome sequencing.

*S. rimosus* ATCC 10970 was sequenced using a hybrid approach combining three next-generation sequencing platforms to balance their shortcomings and strengths; the Illumina GAIIx sequencer was used for obtaining short-read sequences of high quality, and the Pacific Biosciences RS II (PacBio) sequencer was used for obtaining the long reads needed for tackling repetitive regions scattered throughout the genome. These were used together for genome sequencing, while Oxford Nanopore technology was solely used for the sequencing of the plasmids.

Short-read sequencing was performed at Macrogen, Inc. (Daejeon, Republic of Korea). At least 1 μg gDNA was used to construct the short-read genome-sequencing library with the TruSeq DNA PCR-fREE LT kit (Illumina, Inc., San Diego, CA, USA) according to the manufacturer’s instruction.

Long-read genome sequencing for strain ATCC 10970 was performed with PacBio technology at Macrogen, Inc. (Daejeon, Republic of Korea). At least 2 μg gDNA was used as input for PacBio genome sequencing library preparation. The sequencing library was prepared according to a guide for preparing 20-kb SMRTbell template prep kit (Pacific Biosciences, Menlo Park, CA, USA). The templates were sequenced using SMRT sequencing. Library preparation and its quality control were performed at Macrogen, Inc.

### Genome assembly.

The quality of the obtained raw sequencing data was assessed using FastQC (ver. 0.11.5) (53, 54). Given the size of the data set and the relatively high coverage, we ran multiple independent assemblies based on subsets of the reads (subsampling) using seqtk (55), which were further filtered and trimmed using Trimmomatic (ver. 0.36) (56) in order to balance different coverage data sets. For the genome assembly, we ran multiple software assembly pipelines independently, including SPAdes (57), Velvet (ver. 1.2.10) (58), SOAPdenovo (ver. 2.04-r240) (59), and Canu (ver. 1.6) (60), optimizing assembly by testing combinations of multiple parameters such as k-mer, seed size, trimming, etc. Assembled genome quality was assessed with QUAST (ver. 4.4) (61). All computation was performed on AWS r4.8xlarge (32 cores, 244 GB of RAM, 1 TB of hard drive space) and on a local workstation with 32 GB of RAM and with a Xeon E5-1650 ver. 3 multicore processor. Best assembly result was obtained with the combination of hybrid SPAdes and Canu.

### Genome validation: by pulsed-field gel electrophoresis.

For the analysis of the total DNA by pulsed-field gel electrophoresis (PFGE), *S. rimosus* ATCC 10970 genomic DNA was prepared by inoculation, applying a plug of sporulating colonies on MS medium into 5 mL TSB medium, followed by incubation at 28°C with 220 rpm shaking overnight. Preparation of DNA blocks from *S. rimosus* ATCC 10970 was performed as previously described (51). The blocks were treated with AseI or DraI restriction endonuclease (1 block in 400 μL 1× fast digest buffer and 3 μL of enzyme) and incubated overnight to avoid partial digestion. PFGE was performed in a CHEF mapper apparatus (Bio-Rad, USA) in 100 mL 1% low-melting-point agarose gel run in 0.5× TBE buffer (10× TBE buffer: 108 g Tris base, 55 g boric acid, and 40 mL EDTA, pH 8, dissolved in 1 L deionized H_2_O). The gels were stained with ethidium bromide (10 μL of 10 mg/mL ethidium bromide solution in 200 mL distilled water [dH_2_O]) for 20 min, washed twice with 200 mL dH_2_O, and gel image exposed by the GEL Doc instrument. *In silico* virtual gel simulations were made using Geneious (ver. 11.1.5) (62).

### Pulsed-field gel electrophoresis for plasmid extraction.

For the extraction of the plasmid DNA of *S. rimosus* ATCC 10970, M4018, and R6-500, the PFGE method was used to separate plasmid DNA from genomic DNA. A plug of sporulating colonies on MS medium was inoculated into 5 mL TSB medium and incubated at 28°C with 220 rpm shaking overnight. From overnight culture, the blocks containing total DNA were prepared as previously described (51). After the final washing steps, the blocks were stored at 4°C in 0.5 M EDTA without any further treatment. The blocks were run on PFGE gel as described above in “Genome Validation by Pulsed-Field Gel Electrophoresis.” PFGE blocks without restriction enzyme treatment were applicable for plasmid DNA separation on PFGE gel. The plasmid DNA of *S. rimosus* ATCC 10970, M4018, and R6-500 was cut from agarose gel and purified with the E.Z.N.A. gel extraction kit (Omega Bio-tek, USA) following the manufacturer’s instructions. To obtain a sufficient concentration of the plasmid DNA, DNA from at least 10 lanes was cut out and combined before being loaded into a silica column for further purification. The quality and quantity of extracted plasmid DNA was assessed by agarose gel electrophoresis.

### Oxford Nanopore sequencing of plasmids and *de novo* assembly.

The total amount of PFGE-isolated plasmid DNA was used for library construction following the procedure of the ligation sequencing kit (SQK-LSK109, Nanopore Technologies) with a washing step using long fragment buffer (LFB) to enrich longer DNA fragments. The constructed library was loaded either on the preloaded (24 h), washed (flow cell wash kit, EXP-WSH003) MinIon flow cell R9 (samples M4018 and R6-500) or Flongle flow cell (sample ATCC 10970). The cells were run for 20 h with electrical voltage parameters set to −210 mV (preloaded MinIon flow cells) or with default parameters (Flongle flow cell) with MinKNOW software (63). The resulting FAST5 files were base called with Guppy base calling software (ver. 4.2.2). Control lambda phage reads were removed with NanoLyse script (64). Base called sequences were used for *de novo* assembly using the hierarchical Canu assembly pipeline (ver. 2.1.1) (60) or Flye (ver. 2.6) as an alternative (65).

### Genome annotation and secondary metabolite biosynthetic gene cluster prediction.

The complete genome and plasmid sequences were submitted to the NCBI GenBank database and annotated with the NCBI Prokaryotic Genome Annotation Pipeline (PGAP) (annotation software revision v4.1) (66) and are available under RefSeq accession number GCF_006229535.1. After GenBank submission we reannotated the sequences using the local PGAP (ver. 4.3) and manually curated the annotations (modified annotations for 65 genes: [i] OTC cluster genes and OTC resistance genes, [ii] orthologs of Streptomyces coelicolor regulatory genes with a well-defined role in the secondary metabolism, and [iii] orthologs of S. coelicolor Pho regulon genes; full list is in Table S9). Annotations of plasmids M4018 and R6-500 were performed based on the percentage of nucleotide identity (over 90% identity) with ATCC 10970 used as the reference, using Geneious Prime (ver. 2020/2021) (62). The final annotation and assembly were stored on MORF (67). Using the GenBank formatted files, we predicted and analyzed putative biosynthetic gene clusters (BGCs) for secondary metabolites with the Web-based secondary metabolite finder antiSMASH 6.0 (21). The obtained putative BGCs were manually curated to ensure optimized prediction of gene cluster borders. For this downstream analysis of BGCs, we used BLAST (68) and the MIBiG database (69). For protein alignment, Geneious (ver. 11.1.5) and Geneious Prime (ver. 2020/2021) were used (62). The main genomic features, including distribution of rRNA operons, tRNAs, BGCs, and CDSs according to direction of transcription as well as GC-skew diagram and plasmid rearrangements, were visualized using Circa (ver. 1.2.2; OMGenomics).

### Data availability.

Sequence data from ATCC 10970 genome sequencing are available at GenBank (accession no. GCF_006229535.1) and reannotated on MORF (https://morf-db.org/projects/TOPCAPI/streptomyces-rimosus). Sequence data from Nanopore sequencing of isolated plasmids from strains ATCC 10970, M4018, and R6-500 are available at the SRA (no. PRJNA731353). Assembly of plasmids from strains M4018 and R6-500 is available at GenBank (accession no. MZ502218 and MZ502219, respectively). Additional tables and figures, and methods for secondary metabolite analysis in Streptomyces rimosus ATCC 10970, are available in supplemental files.
