# Supplementary material for: Reference-Grade Genome and Large Linear Plasmid of Streptomyces rimosus: Pushing the Limits of Nanopore Sequencing
Source: Microbiol Spectr. 2022 Apr 4;10(2):e02434-21. doi: 10.1128/spectrum.02434-21 (PMC9045324; doi:10.1128/spectrum.02434-21)
Supplement: SUPPLEMENTAL FILE 1 — Supplemental material. Download SPECTRUM02434-21_Supp_1_seq11.pdf, PDF file, 0.7 MB [file spectrum02434-21_supp_1_seq11.pdf]

Reference grade genome and large linear plasmid of *Streptomyces rimosus* - pushing the limits of Nanopore sequencing

Lucija Slemc[a], Jernej Jakše[b], Alessandro Filisetti[c], Damir Baranasic[d], Antonio Rodríguez García[e], Francesco Del Carratore[f], Jurica Zucko[d], Antonio Starcevic[d], Martin Šala[g], Mercedes Pérez-Bonilla[h], Marina Sánchez-Hidalgo[h], Ignacio González[h], Fernando Reyes[h], Olga Genilloud[h], Vicki Springthorpe[i], Dušan Goranovič[j], Gregor Kosec[j], Gavin H. Thomas[i], Davide De Lucrezia[c], Hrvoje Petković[a], Miha Tome[a]#

[a]Food Science and Technology Department, Biotechnical Faculty, University of Ljubljana, Jamnikarjeva 101, 1000 Ljubljana, Slovenia;

[b]Department of Agronomy, Biotechnical Faculty, University of Ljubljana, Jamnikarjeva 101, 1000 Ljubljana, Slovenia;

[c]Explora Biotech Srl, Doulix business unit, Via Torino 107, 30133 Venice, Italy;

[d]Faculty of Food Technology and Biotechnology, University of Zagreb, Pierottijeva 6, HR-10000 Zagreb, Croatia;

[e]INBIOTEC Instituto de Biotecnología de León, Parque Científico de la Granja, Avenida Real 1, 24006 León, Spain;

[f]Manchester Institute of Biotechnology, Faculty of Science and Engineering, University of Manchester, Manchester, United Kingdom;

[g]National Institute of Chemistry, Hajdrihova 19, SI-1000 Ljubljana, Slovenia;

[h]Fundación MEDINA, Parque Tecnológico Ciencias de la Salud. Avda. del Conocimiento 34, 18016 Granada, Spain;

[i]Department of Biology, University of York, Wentworth Way, York, YO10 5DD, United Kingdom;

[j]Acies Bio d.o.o., Tehnološki Park 21, 1000, Ljubljana, Slovenia

Running Head: *S. rimosus* genome; pushing the limits of Nanopore seq.

#Address correspondence to Miha Tome, miha.tome@bf.uni-lj.si

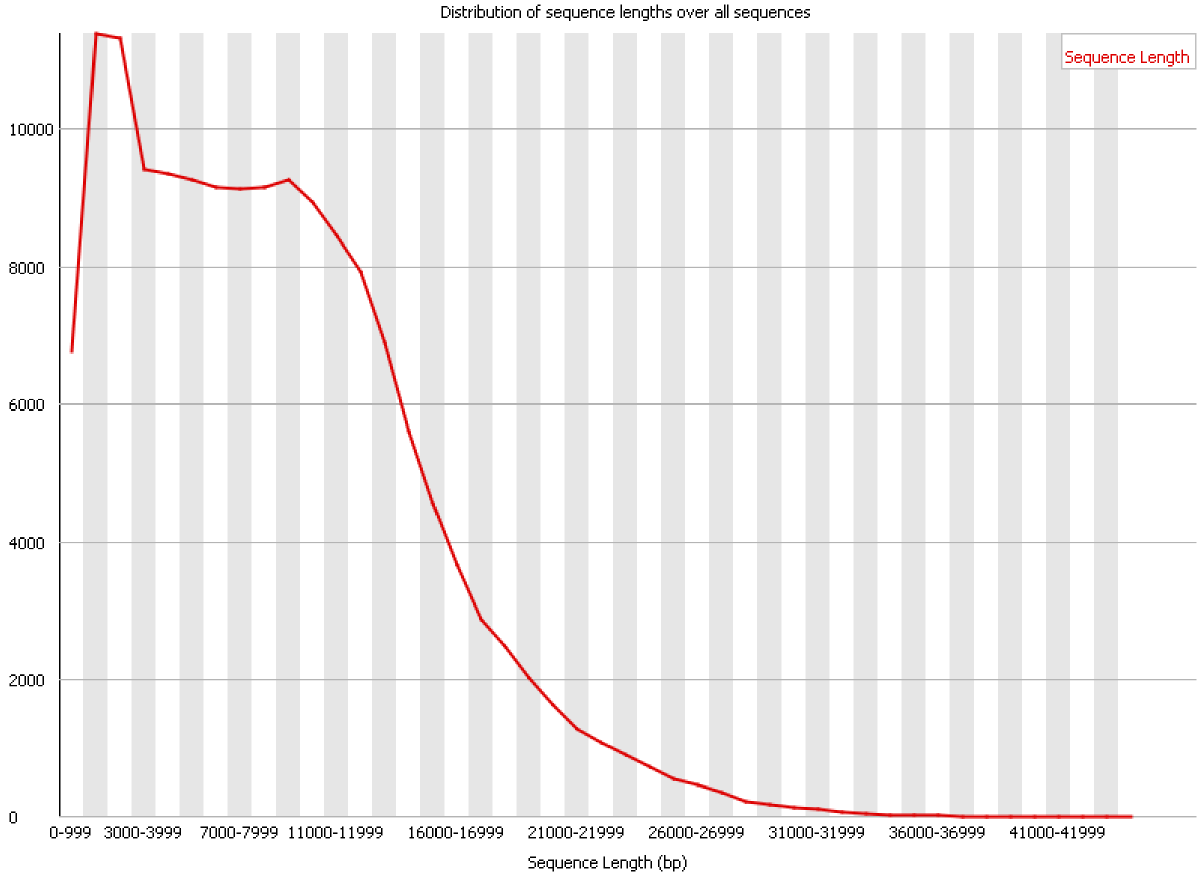

Figure S1: Distribution of sequence lengths of PacBio sequencing of genomic DNA from *S. rimosus* ATCC 10970.

Table S1: *S. rimosus* gene space completeness of *S. rimosus* genome assemblies found on RefSeq NCBI.

Strain	Complete and single-copy	Complete and duplicated	Fragmented	Missing	Total	Gene space completeness (%)	Accession number

**NRRL 2234 / ATCC 10970**	**1569**	**5**	**3**	**2**	**1579**	**99.7**	**GCF_006229535.1**
NRRL 2234	1568	5	3	3	1579	99.6	GCF_008704655.1
M527	1417	7	6	149	1579	90.1	GCF_004196335.1
NBRC 15454	1565	7	5	2	1579	99.5	GCF_003865155.1
NRRL WC-3897	1566	4	4	5	1579	99.5	GCF_001507395.1
NRRL WC-3898	1563	4	9	3	1579	99.3	GCF_001279375.1
NRRL WC-3896	1563	4	7	5	1579	99.3	GCF_001279345.1
NRRL WC-3873	1567	4	5	3	1579	99.5	GCF_001279105.1
NRRL WC-3899	1566	5	5	3	1579	99.5	GCF_001279095.1
NRRL WC-3875	1568	4	5	2	1579	99.6	GCF_001279075.1
NRRL WC-3869	1566	4	5	4	1579	99.5	GCF_001279065.1
NRRL WC-3909	1565	3	7	4	1579	99.3	GCF_001279015.1
NRRL WC-3929	1560	7	4	8	1579	99.2	GCF_000721665.1
NRRL B-2626	1569	4	3	3	1579	99.7	GCF_000721045.1
NRRL WC-3904	1556	6	8	9	1579	98.9	GCF_000720725.1
NRRL WC-3900	1568	4	4	3	1579	99.6	GCF_000720715.1
NRRL WC-3877	1402	5	5	167	1579	89.1	GCF_000720685.1
NRRL WC-3558	1568	5	3	3	1579	99.6	GCF_000720605.1
NRRL WC-3927	1375	2	9	193	1579	87.2	GCF_000720595.1
NRRL WC-3925	1556	4	4	15	1579	98.8	GCF_000720565.1
NRRL B-2660	1567	4	4	4	1579	99.5	GCF_000719185.1
NRRL WC-3880	1398	5	6	170	1579	88.8	GCF_000718895.1
NRRL WC-3882	1511	4	4	60	1579	96.0	GCF_000718865.1
NRRL WC-3876	1522	4	3	50	1579	96.7	GCF_000718835.1
NRRL WC-3560	1499	5	5	70	1579	95.2	GCF_000718755.1
NRRL WC-3874	1565	2	6	6	1579	99.2	GCF_000718715.1
NRRL WC-3930	1539	8	6	26	1579	98.0	GCF_000718675.1
NRRL WC-3924	1565	3	4	7	1579	99.3	GCF_000717815.1
NRRL ISP-5260	1570	4	3	2	1579	99.7	GCF_000717285.1
NRRL B-8076	1414	4	6	155	1579	89.9	GCF_000716745.1
NRRL B-16073	1568	4	3	4	1579	99.6	GCF_000716515.1
R6-500MV9-R8	1541	1	15	22	1579	97.7	GCF_000707965.1
R6-500MV9	1557	1	5	16	1579	98.7	GCF_000707945.1
R6-500	1567	3	3	6	1579	99.4	GCF_000707925.2
NRRL 2234	1569	4	3	3	1579	99.7	GCF_000331185.2

Assembly completed in this study is highlighted with bold – with accession number GCF_006229535.1.

Table S2: Assemblies for ATCC 10970.

	Total length (bp)	#contig	#contig 1000	Largest contig	N50	L50	GC (%)
**SPAdes Hybrid**	9,711,078	1,289	15	9,358,594	9,358,594	1	71.7
**CANU**	9,654,011	3	3	9,363,743	9,363,743	1	71.9
**VELVET**	9,602,288	7,754	229	622,933	304,166	11	71.7
**SOAP**	8,972,304	783	131	622,816	170,464	18	71.9

#contig = number of contigs; #contig 1000 = number of contigs bigger than 1000 base pairs, bp; Largest contig = size (in bp) of the biggest contig; N50 = the shortest sequence length of contigs covering 50% of the genome; L50 = smaller number of contigs covering 50% of the genomes; GC (%) = relative GC content. Hybrid assembling refers to the fact that both Illumina and PacBio reads were used.

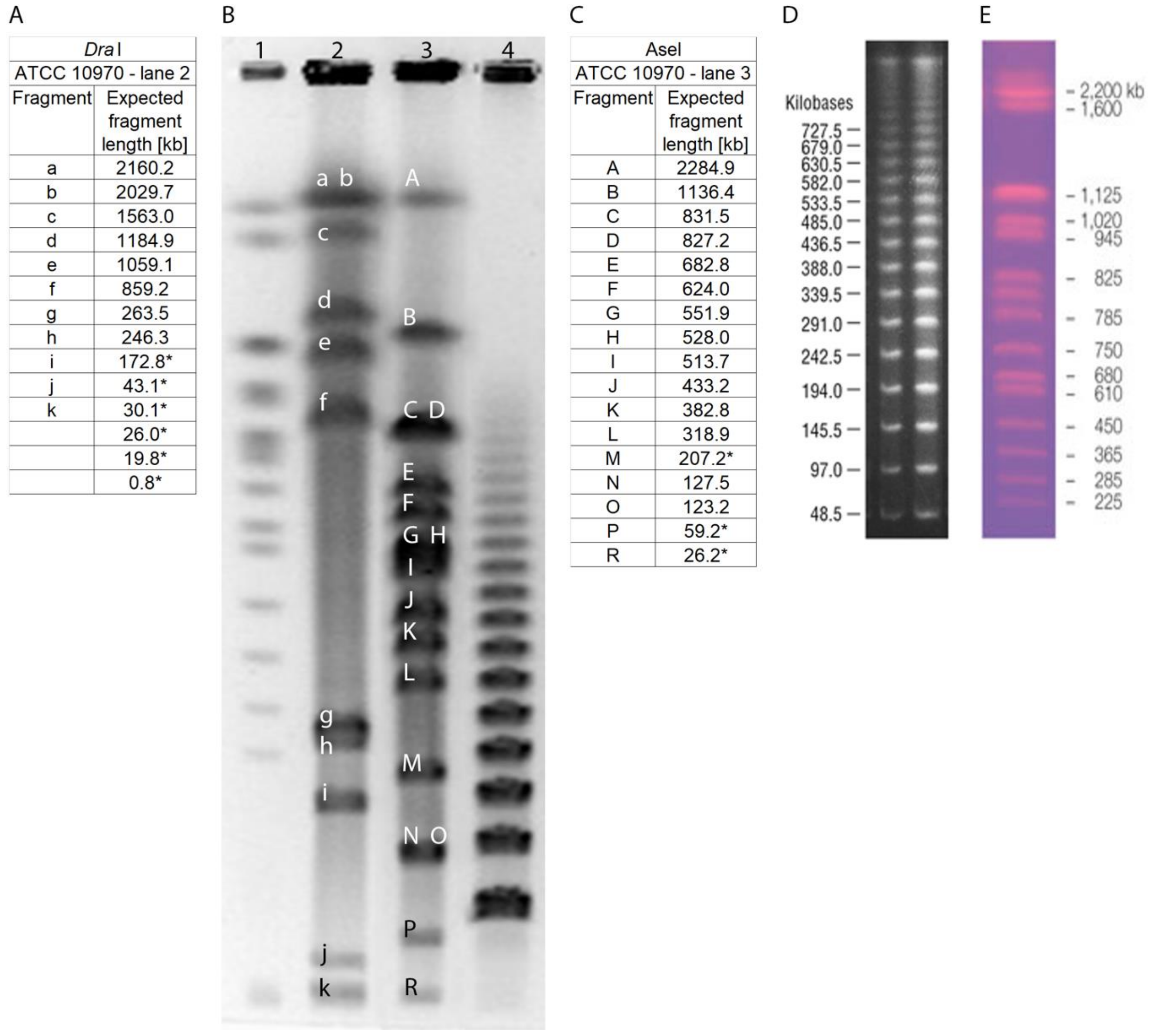

Figure S2: Genome analysis of *S. rimosus* ATCC 10970 by pulsed-field gel electrophoresis (PFGE) compared to *in silico* total DNA digestion. **A)** *In silico* estimated DNA sizes of *Dra*I fragments of total *S. rimosus* ATCC 10970. **B)** PFGE of DNA samples prepared in gels and digested with either *Dra*I or *Ase*I. Lanes: 1. *S. cerevisiae* chromosomal DNA PFGE marker; 2. *S. rimosus* ATCC 10970 total DNA digested with *Dra*I; 3. *S. rimosus* ATCC 10970 total DNA digested with *Ase*I; 4. λ PFG ladder marker. **C)** *In silico* estimated DNA sizes of *Ase*I fragments of total *S. rimosus* ATCC 10970 DNA. **D)** λ PFG ladder marker (New England BioLabs) **E)** *S. cerevisiae* chromosomal DNA PFGE marker (Bio Rad). The asterisk marks DNA fragments derived from digestion of plasmid DNA.

Table S3: List of the additional CDSs found in the M4018 plasmid transferred from the chromosome.

Locus tag ATCC 10970	Locus tag M4018	Strand	Product
WT5260_052820	M4018_052820	+	hypothetical protein
WT5260_052830	M4018_052830	+	chitin-binding protein
WT5260_052840	M4018_052840	+	GAF and ANTAR domain-containing protein
WT5260_052850	M4018_052850	-	hypothetical protein
WT5260_052860	M4018_052860	-	ATP-binding protein
WT5260_052870	M4018_052870	-	Mu transposase C-terminal domain-containing protein
WT5260_052880	M4018_052880	-	TnsA-like heteromeric transposase endonuclease subunit

Table S4: List of mutations identified when comparing the M4018 plasmid with the ATCC 10970 plasmid.

Mutation	position ATCC 10970	position M4018	Note
g->t	14686	14726	M4018_081910*
a->c	14687	14727	M4018_081910*
Δ1 bp	14688	14727	M4018_081910†
Δ1 bp	32720	32757	M4018_082090// M4018_082100
c->t	53730	53768	M4018_082330
c->a	109202	109240	M4018_082990*
Δ1 bp	186510	186547	M4018_083730†
a->t	198389	198426	M4018_083840*
a->g	198392	198429	M4018_083840*

Δ5 bp	204859	204896	M4018_052820// M4018_083910
c->t	220770	227510	M4018_084000*
Δ4 bp	251044	257780	M4018_084240// M4018_084250

Table S5: List of mutations identified when comparing the R6 plasmid with the ATCC 10970 plasmid.

Mutation	Position ATCC 10970	Position M4018	Note
+g	29913	29958	R6500_082050†
+gg	29971	30017	R6500_082050†
+g	32684	32732	R6500_082090 // R6500_082100
Δ3 bp	32708	32757	R6500_082090 // R6500_082100
+27 bp	32711	32758	R6500_082090 // R6500_082100
+t	32786	32859	R6500_082090 // R6500_082100
c->t	98858	98858	R6500_082850
Δ3 bp	115426	115500	R6500_083070†
+54 bp	115429	115501	R6500_083070†
+c	115458	115583	R6500_083070†
Δ1 bp	157429	157555	R6500_083410†
Δ1 bp	157447	157572	R6500_083410†
Δ1 bp	162974	163098	R6500_083460†
Δ2 bp	163261	163384	R6500_083470†

Table S6: List of CDSs present in the rearranged section of the R6 sequence.

Locus tag ATCC 10970	Locus tag R6	Strand	Product
WT5260_083830	R6500_083560	-	IS5 family transposase
WT5260_083820	R6500_083570	-	hypothetical protein
WT5260_083810	R6500_083580	-	hypothetical protein
WT5260_083800	R6500_083590	-	Transposase
WT5260_083780	R6500_083600	+	S1 RNA-binding domain-containing protein
WT5260_083790	R6500_083610	+	IS5 family transposase
WT5260_083800	R6500_083620	+	Transposase
WT5260_083810	R6500_083630	+	hypothetical protein
WT5260_083820	R6500_083640	+	hypothetical protein
WT5260_083830	R6500_083650	+	IS5 family transposase
WT5260_083550	R6500_083660	+	IS5 family transposase
WT5260_083540	R6500_083670	+	IS701 family transposase
WT5260_083530	R6500_083680	+	hypothetical protein
WT5260_083520	R6500_083690	-	hypothetical protein

WT5260_083510	R6500_083700	-	hypothetical protein
WT5260_083500	R6500_083710	-	hypothetical protein
WT5260_083490	R6500_083720	+	hypothetical protein
WT5260_083480	R6500_083730	-	hypothetical protein
WT5260_083470	R6500_083740	-	restriction endonuclease
WT5260_083460	R6500_083750	+	helix-turn-helix transcriptional regulator
WT5260_083450	R6500_083760	+	HD domain-containing protein
WT5260_083440	R6500_083770	-	relaxase domain-containing protein
WT5260_083430	R6500_083780	+	hypothetical protein
WT5260_083420	R6500_083790	-	cytochrome P450

Table S7: Putative biosynthetic gene cluster on plasmids of *S. rimosus* ATCC 10970, M4018 and R6-500.

Cluster No. in ATCC 10970	Type	From	To	Most similar known biosynthetic gene cluster (percent of similarity)	Location in M4018 plasmid		Location in R6 plasmid	
1	Type I PKS	143989	163050	kanamycin (1 %)	144008	163069	143937	162984
2	NRPS	215829	230795	NA	215875	230814	/	/

Table S8: Coding sequences at regions where contigs are not connected with each-other in the mapping of genome assembly GCF_000716745.1 to our GCF_006229535.1 plasmid sequence.

Product	Length [bp]	Direction	Start	End	Locus tag
IS701 family transposase	576	reverse	166,822	167,397	WT5260_083540
IS5 family transposase	843	reverse	167,447	168,289	WT5260_083550
IS5 family transposase	867	forward	196,040	196,906	WT5260_083830
IS5 family transposase	843	forward	197,573	198,415	WT5260_083840
IS5 family transposase	870	forward	198,701	199,570	WT5260_083850
transposase	837	forward	199,618	200,454	WT5260_083860
IS481 family transposase	873	forward	241,050	241,922	WT5260_084170
transposase	655	forward	260,815	261,469	WT5260_084320
IS5/IS1182 family transposase	768	reverse	261,700	262,467	WT5260_084330

Cultivation conditions and extraction of secondary metabolites from *S. rimosus* ATCC 10970

For identification of different secondary metabolites produced by *S. rimosus* ATCC 10970 we have used different mediums and solvents for extraction. *S. rimosus* was inoculated into GOTCV seed medium at 30 °C, 220 rpm for 24 h. 10% (v/v) of seed medium was used to inoculate in GOTC production medium (1, 2) and cultivated for 5 days at 30 °C, 220 rpm and 60 % humidity. Additionally, we used ISP2 as seed medium and ISP4 as production medium (3) and cultivation was performed under same conditions as for GOTC media. For detection of oxytetracycline, rimocidin, antibiotic CE108, antibiotic CE 108 amide, rimosamides and guanipiperazines the pH of culture broth was adjusted to pH 2 and extracted with acetonitrile. Metabolites ectoin, cyhmostatin and tyrobetaines were extracted from mycelium using methanol:acetone (1:1). Organic solvent was evaporated, and dry extracts dissolved in methanol for LC-MS analysis. For pseudouridimycin detection culture broth was centrifuged and the supernatants filtered before LC-MS analysis.

LC-MS analysis and dereplication of extracts

For production of oxytetracycline, rimocidn, CE108 and rimosamides LC-MS analysis were performed on UltiMate 3000 UHPLC system (Thermo Scientific, U.S.A.) coupled with a triple quadrupole/linear ion trap mass spectrometer (4000 QTRAP LC-MS/MS System; Applied Biosystems/MDS Sciex, Ontario, Canada). Methanol (Chromasolv LC-MS grade, Fluka, Switzerland) and water purified on a Milli-Q system from Millipore (Bedford, MA, USA) were used for the preparation of mobile phases, formic acid from Fluka was used as modifier. An analytical HPLC column Kinetex XB-C18 100A (3.0 × 150 mm, 3 µm particle size, Phenomenex) was used with the flow rate of 0.3 mL min-1. A mobile phase consisted of acetonitrile and water, both modified with 0.1% formic acid was used throughout the work. Injection volume and column temperature were 10 µL and 30 °C, respectively. HRMS measurements were performed with a hybrid quadrupole orthogonal acceleration time-of-flight mass spectrometer (QTOF Premier, Waters, Milford, MA, U.S.A.).

Another LC-HRMS analysis was performed on an Agilent 1100 single quadrupole LC-MS system, using an Atlantis T3 column (5 µm, 4.6 × 100 mm), maintained at 40 °C and with a flow rate of 1 mL min^{-1}. Solvent A consisted of 0.1% HCOOH in water and solvent B was 0.1% HCOOH in acetonitrile. The elution of solvent B started at 5% for 2 min and was increased to 100% within 11 min, this composition was maintained for 3 min, after which the percentage of B was decreased to 5% within 1 min. To re-equilibrate the system, the elution of B was held at 5% for 3 min. HRESIMS spectra were acquired using a Bruker maXis QTOF mass spectrometer coupled to the same HPLC system as described above. The mass spectrometer was operated in positive ESI mode. The instrumental parameters were 4 kV capillary voltage, drying gas flow of 11 L min^{-1} at 200 °C, and nebulizer pressure of 2.8 bar.

The dereplication of the known compounds (oxytetracycline, antibiotic CE 108, antibiotic CE 108 amide, rimocidin, etc.) were performed by comparison of the molecular formulae and UV spectra of the components detected with the Dictionary of Natural Products database. Figure S3 shows the LC-UV chromatographic profile at 275 nm of the acetonitrile extract and table S9 includes the LC-HRMS data of dereplicated compounds. The UV and MS spectra of the major and minor compounds are shown in figures S4-S8. 2-Acetyl-2-decarboxamido-oxytetracycline (ADOTC) and rimosamides A-D were detected at trace level and were putatively identified.

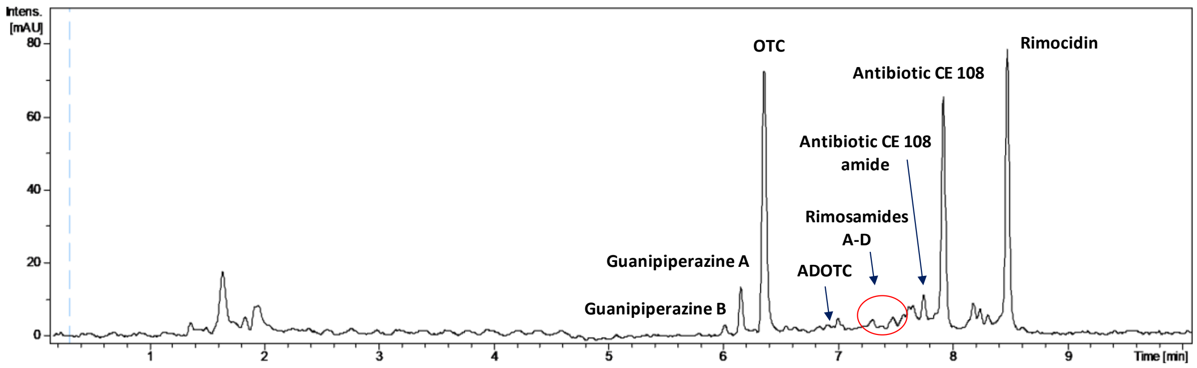

Figure S3: LC-UV profile of the acetonitrile extract from *S. rimosus* ATCC 10970 culture at 275 nm.

Table S9: LC-HRMS data of the dereplicated compounds.

RT (min)	$[M+H]^+$	*m/z*	MF	Compound
**6.00**	446.1927	223.5997 ($[M+2H]^{2+}$)	C23H23N7O3	Guanipiperazine B
**6.15**	446.1927	223.6001 ($[M+2H]^{2+}$)	C23H23N7O3	Guanipiperazine A

**6.35**	461.1552		$C_{22}H_{24}N_2O_9$	OTC
**6.81**	460.1590		$C_{23}H_{25}NO_9$	ADOTC
**7.21**	533.2928		$C_{27}H_{40}N_4O_7$	Rimosamide D
**7.37**	591.3013		$C_{29}H_{42}N_4O_9$	Rimosamide B
**7.38**	547.3120		$C_{28}H_{42}N_4O_7$	Rimosamide C
**7.52**	605.3181		$C_{30}H_{44}N_4O_9$	Rimosamide A
**7.74**	739.4003	761.3824 ($[M+Na]^+$)	$C_{37}H_{58}N_2O_{13}$	Antibiotic CE 108 amide
**7.90**	740.3847		$C_{37}H_{57}NO_{14}$	Antibiotic CE 108
**8.46**	768.4161		$C_{39}H_{61}NO_{14}$	Rimocidin

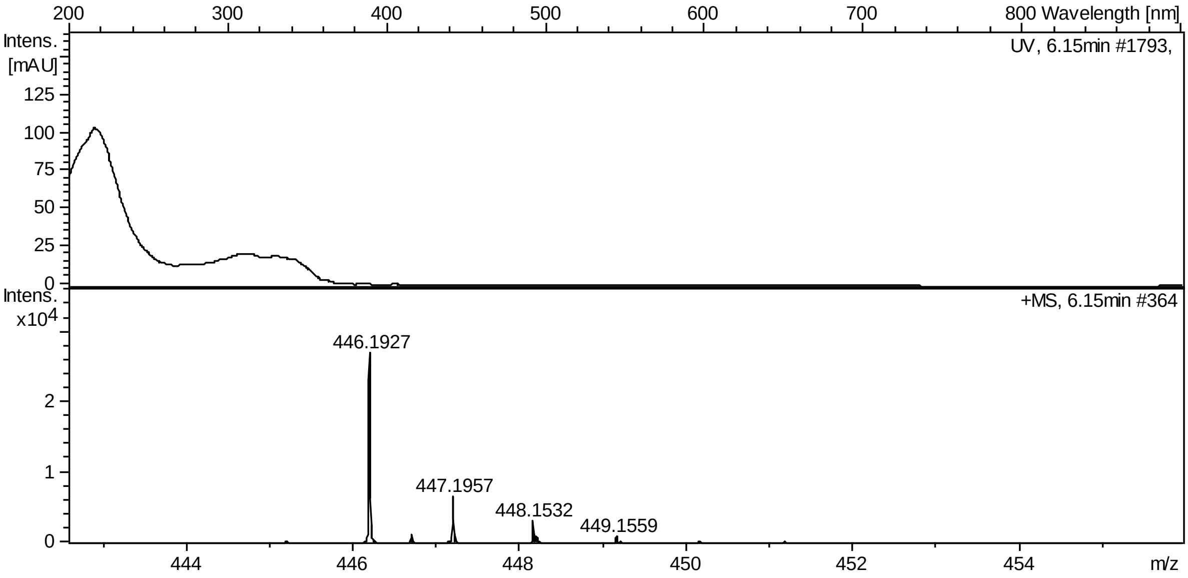

Figure S4: UV and HRMS spectra of guanipiperazines.

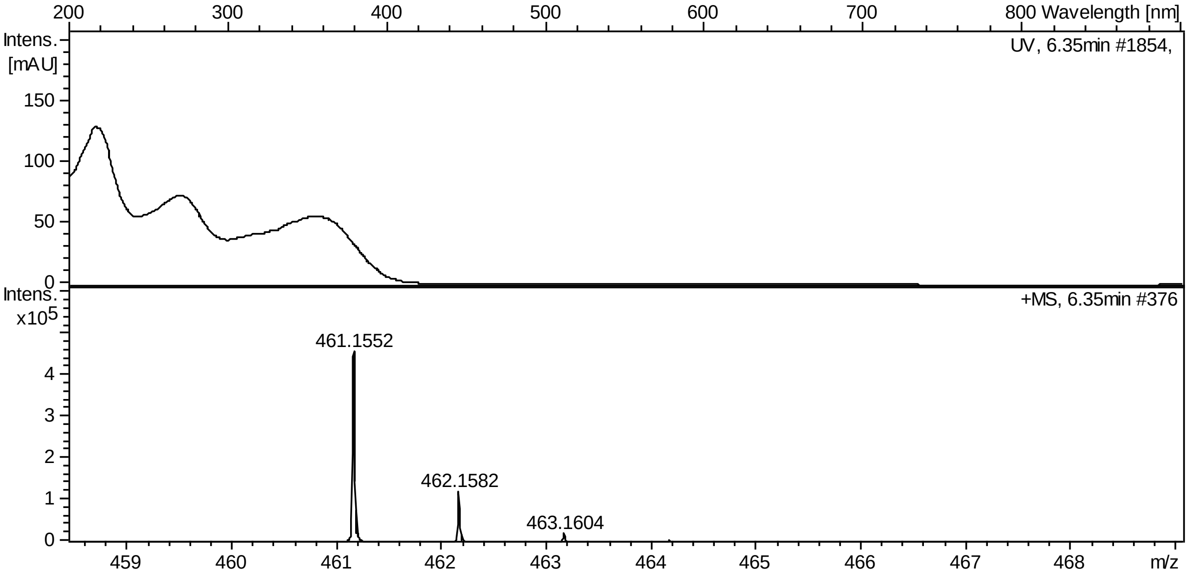

Figure S5: UV and HRMS spectra of oxytetracycline.

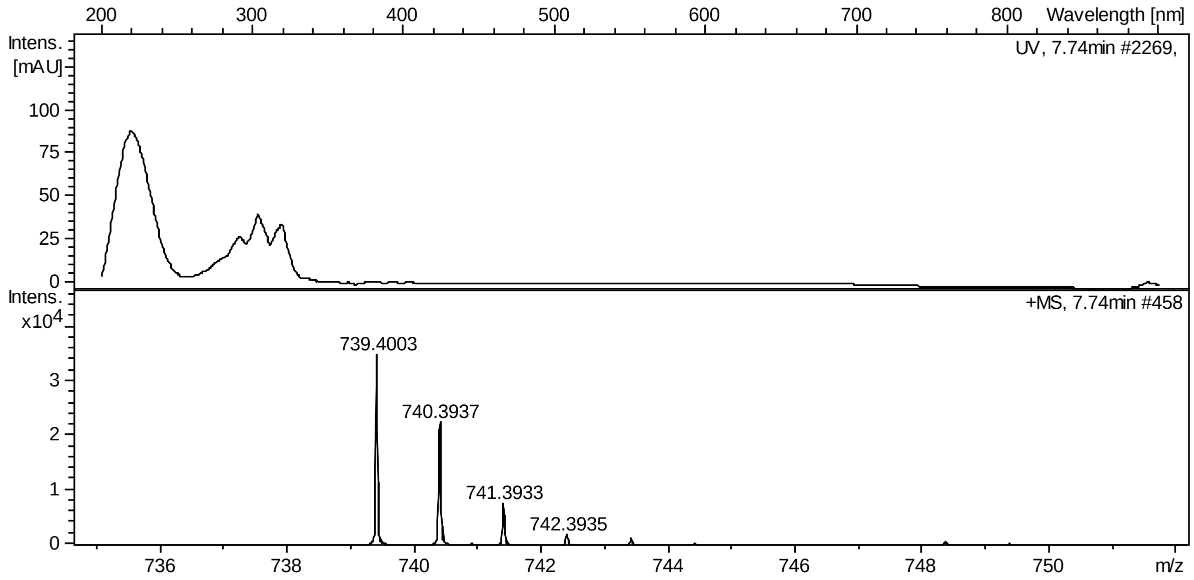

Figure S6: UV and HRMS spectra of antibiotic CE 108 amide.

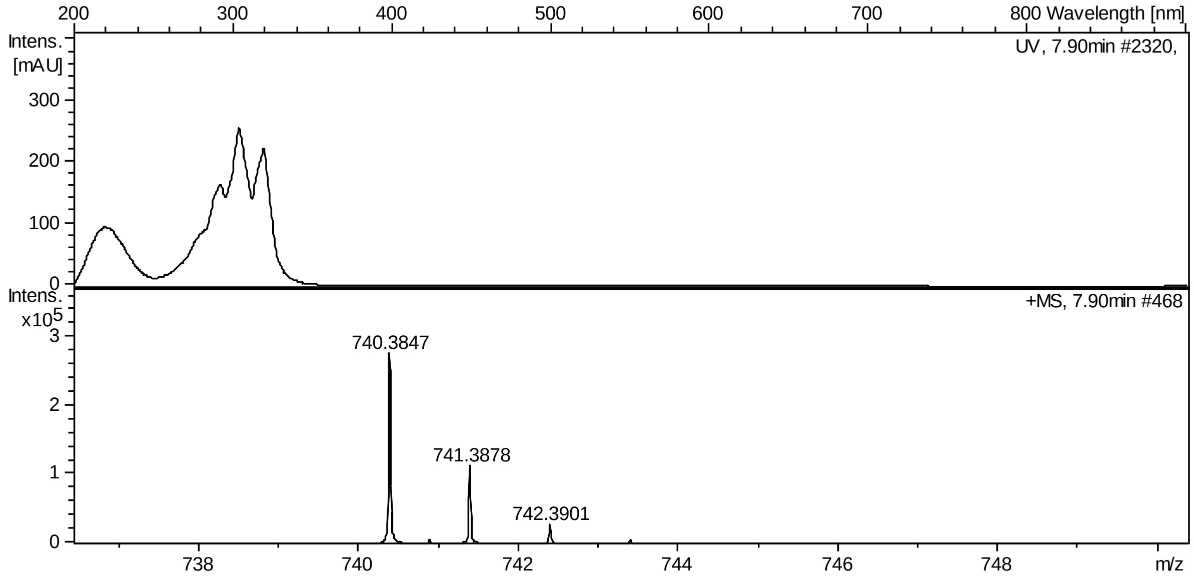

Figure S7: UV and HRMS spectra of antibiotic CE 108.

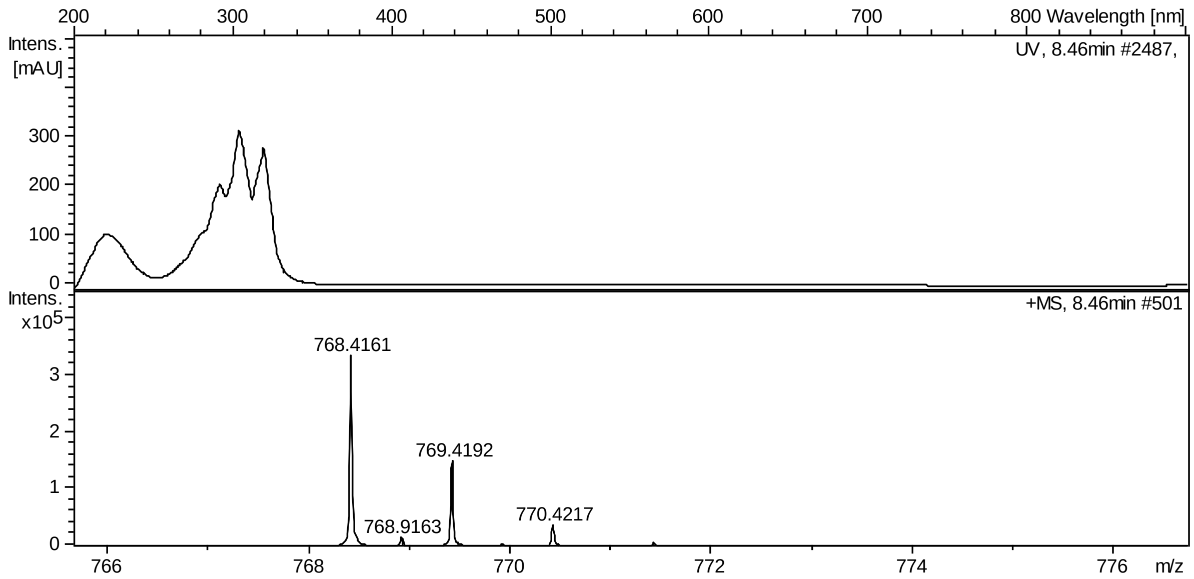

Figure S8: UV and HRMS spectra of rimocidin.

For production of tyrobetaines, ectoine, hydroxyectoine, chymostatin and pseudouridimycin LC-MS analysis was performed on UltiMate 3000 UHPLC system (Thermo Scientific, U.S.A.) coupled with a triple quadrupole/linear ion trap mass spectrometer (4000 QTRAP LC-MS/MS System; Applied Biosystems/MDS Sciex, Ontario, Canada). Methanol (Chromasolv LC-MS grade, Fluka, Switzerland) and water purified on a Milli-Q system from Millipore (Bedford, MA, USA) were used for the preparation of mobile phases, formic acid from Fluka was used as modifier. An analytical HPLC column Kinetex XB-C18 100A (3.0 × 150 mm, 3 µm particle size, Phenomenex) was used with the flow rate of 0.3 mL min^{-1}. A mobile phase consisted of water (mobile phase A) and acetonitrile (mobile phase B), both modified with 0.1 % formic acid was used throughout the work. Injection volume and column temperature were 10 µL and 30 °C, respectively. The following gradient was used:

Time [min]	% of A	% of B
0	95	5
15	55	45
15.5	10	90
18	10	90
18.1	95	5
23	95	5

Samples were extracted with different solvents as described in previous paragraph and were screened with a wide m/z range Q1 method. We have then examined the results of the XIC (extracted ion chromatogram) and identified masses that could be

connected to individual metabolites. For the promising leads of the individual compound peaks a MS^2 experiment was set-up. These fragmentation patterns were then compared to literature data. This approach was used to confirm production of tyrobetaines. To confirm biosynthesis of ectoine, hydroxyectoine, chymostatin and pseudouridimycin in *S. rimosus* we have used standards obtained by Sigma-Aldrich and pseudouridimycin was kindly provided by Naicons Srl. LC-MS method for conformation of pseudouridimycin method was slightly modifies as in Sosio *et al*. (4).

Table S10: Selected reaction monitoring (SRM) experiment.

	Transition	Declustering potential (DP)	Collision energy (CE)
Ectoine	143 → 68	50	39
	143 → 95	50	22
Hydroxyectoine	159 → 113	73	21
	159 → 83	73	34
Chymostatin A, C	608 → 449	120	39
	608 → 417	120	37
Chymostatin B	594 → 429	150	35
	594 → 403	150	37
Pseudouridimycin	487 → 371	55	30
	487 → 217	55	35

General experimental procedures

Optical rotations were measured using a Jasco P-2000 polarimeter (JASCO Corporation, Tokyo, Japan). UV spectra were obtained with an Agilent 1100 DAD (Agilent Technologies, Santa Clara, CA, USA). IR spectra were recorded on a JASCO FT/IR-4100 spectrometer (JASCO Corporation, Tokyo, Japan) equipped with a PIKE MIRacle™ single reflection ATR accessory. NMR spectra were recorded on a Bruker Avance III spectrometer (500 and 125 MHz for ^{1}H and ^{13}C NMR, respectively) equipped with a 1.7 mm TCI MicroCryoProbe™ (Bruker Biospin, Fälländen, Switzerland). Chemical shifts were reported in ppm using the signals of the residual solvent as internal reference (δ_H 2.50 and δ_C 39.51 for DMSO-d_6).

Extraction and Isolation Guanipiperazines A and B

The fermentation pH was adjusted below 2 with 37% HCl before extracting with acetonitrile in the fermentation flasks (1:1, 50 mL), in an orbital shaker at 220 rpm for 1 h. After centrifugation (5 min, 8500 rpm) and filtration under vacuum. acetonitrile was

evaporated under a nitrogen stream to reach the original fermentation volume (1 L) and the aqueous extract that was loaded onto an SP207ss resin column (65 g, 32 × 100 mm) and fractionated with an acetone−H_2O stepped gradient (10/90 for 6 min, 20/80 for 6 min, 40/60 for 6 min, 60/40 for 6 min, 80/20 for 6 min, and 100/0 for 12 min, 10 mL/min, 20 mL/fraction) in a Teledyne CombiFlash RF apparatus. Fractions 12 to 15 were pooled and subjected to preparative reversed-phase HPLC (Atlantis T3, 19 × 250 mm, 5 µm, 14 mL/min, UV detection at 210 and 275 nm, 8.75 mL/fraction) using H_2O + 0.1% TFA (solvent A) and CH_3CN + 0.1% TFA (solvent B). Elution was carried out using isocratic conditions of 5% B for 5 min and then a linear gradient from 5% to 70% B for 52 min, held at 70% B for 8 min, yielding 80 fractions.

Subfraction 27 was further purified by semipreparative reversed-phase HPLC (Atlantis T3, 10 × 150 mm, 5 µm, 3.6 mL/min, UV detection at 210 and 275 nm, 1.8 mL/fraction) using H_2O + 0.1% TFA (solvent A) and CH_3CN + 0.1% TFA (solvent B). Elution was carried out using isocratic conditions of 10% B for 5 min followed by a linear gradient from 10% B to 30% B in 35 min to yield guanipiperazine B (t_R 16.5 min, 1.2 mg).

Subfraction 28 was further purified by semipreparative reversed-phase HPLC (Atlantis T3, 10 × 150 mm, 5 µm, 3.6 mL/min, UV detection at 210 and 275 nm, 1.8 mL/fraction) using H_2O + 0.1% TFA (solvent A) and CH_3CN + 0.1% TFA (solvent B). Elution was carried out using isocratic conditions of 8% B for 5 min followed by a linear gradient from 8% B to 16% B in 35 min to yield guanipiperazine A (t_R 31.0 min, 2.0 mg).
The structures of guanipiperazine A and B were characterized by 1D and 2D NMR data analysis (Table S11).

Guanipiperazine A (**1**): white amorphous solid; $[\alpha]_D^{25}$ – 35.3 (*c* 0.48, MeOH); UV (DAD) λ_{max} 240, 270, 320, 360 nm; IR (ATR) ν_{max} 3379, 1673, 1499, 1432, 1198, 1132, 1018 cm^{-1}; for ^1H and ^{13}C NMR data see Table S11; (+)-ESI-qTOF MS *m/z* 446.1940 $[M+H]^+$ (calcd for $C_{23}H_{24}N_7O_3^+$, 446.1935).

Guanipiperazine B (**2**): white amorphous solid; $[\alpha]_D^{25}$ – 43.1 (*c* 0.50, MeOH); UV (DAD) λ_{max} 240, 280 nm; IR (ATR) ν_{max} 3360, 1673, 1500, 1415, 1197, 1132, 1013 cm^{-1}; for

^{1}H and ^{13}C NMR data see Table S11; (+)-ESI-qTOF MS *m/z* 446.1942 $[M+H]^+$ (calcd for $C_{23}H_{24}N_7O_3^+$, 446.1935).

Table S11: NMR spectroscopic data (500 MHz, DMSO-d_6) for guanipiperazines A and B.

Position	Guanipiperazine A (1)		Guanipiperazine B (2)	
	δ_C, Type	δ_H, Mult. (*J* in Hz)	δ_C, Type	δ_H, Mult. (*J* in Hz)
Piperazinomycin				
2	42.7, CH_2	2.73, brd (12.7)<br>2.39, dd (12.7, 10.7)	42.9, CH_2	2.67, brd (12.6)<br>2.41, dd (12.6, 9.6)
3	51.9, CH	3.02, dd (10.7, 2.7)	52.0, CH	2.95, dd (9.6, 2.5)
4	34.2, CH_2	2.85, dd (17.2, 2.7)<br>2.55, dd (17.2, 3.5)	34.5, CH_2	2.85, dd (17.6, 2.5)<br>2.59, dd (17.6, 3.3)
5	126.7, C		135.6, C	
6	121.9, CH	6.03 brs	121.2, CH	6.24 brs
7	153.9, C		153.6, C	
8	144.6, C		138.6, C	
9	151.4, C		121.7, CH	7.11, d (8.2)
10	119.4, CH	7.21, brs	122.7, CH	6.63, dd (8.2, 1.3)
12	46.8, CH_2	3.48, d (13.3)<br>3.09, dd (13.3, 5.7)	47.1, CH_2	3.43, d (13.0)<br>3.04, dd (13.0, 5.4)
13	48.6, CH	3.63, brd (5.7)	48.8, CH	3.60, brd (5.4)
14	37.7, CH_2	3.28, dd (14.5, 3.2)<br>3.19, dd (14.5, 3.5)	37.8, CH_2	3.26, dd (14.5, 2.9)<br>3.17, dd (14.5, 3.7)
15	135.3, C		136.0, C	
16	132.7, CH	7.59, dd (8.4, 2.1)	132.8, CH	7.58, dd (8.3, 2.1)
17	124.3, CH	6.95, dd (8.4, 2.4)	123.9, CH	6.93, dd (8.3, 2.4)
18	160.5, C		159.6, C	
19	125.7, CH	7.27, dd (8.3, 2.4)	125.3, CH	7.23, dd (8.3, 2.4)
20	132.0, CH	7.65, dd (8.3, 2.1)	132.0, CH	7.64, dd (8.3, 2.1)
Guanine				
21	146.8, C		153.1, C	
22				
23	117.2, C		117.3, C	
24	154.9, C		156.9, C	
25				
26	152.0, C		152.3, C	
27				
28	*		149.5, C	

*Not observed.

Table S12: Closest BLAST homolog for each protein encoded by the *rms* Biosynthetic Gene Cluster (BGC).

Proteins from *rms* Cluster	Closest BLAST Homolog (Organism) Reference (% Identity/% Similarity)	% Identity/similarity with Gup proteins
**RmsA**	Pyridoxamine 5'-phosphate oxidase family protein (*Streptomyces*) WP_003980005.1 (100/100)	77.8/86.4
**RmsB**	Amino acid adenylation domain-containing protein (*Streptomyces*) WP_003980006.1 (100/100)	81.1/86.2
**RmsC**	Cytochrome P450 (*Streptomyces*) WP_003980007.1 (100/100)	87.1/90.9
**RmsD**	Cytochrome P450 (*Streptomyces*) WP_003980008.1 (100/100)	84.1/90.7

Table S13: Proposed genes involved in pseudouridimycin biosynthesis from *S. rimosus* ATCC 10970 and comparison with related genes from *Streptomyces sp*. ID38640.

Locus tag in *S. rimosus* ATCC 10970	Proposed function	Homologous gene in Streptomyces sp. ID38640	Locus tag in Streptomyces sp. ID38640	Amino acid identity/similarity [%]
WT5260_079280	hydroxylation	*pumE*	AVT42374.1	85/90
WT5260_079290	unknown	*pumF*	AVT42375.1	70/77
WT5260_079300	aminotransferase	*pumG*	AVT42376.1	81/88
WT5260_079310	hosphorylation of a uridine-based substrate	*pumH*	AVT42377.1	80/89
WT5260_079320	oxidase	*pumI*	AVT42378.1	78/85
WT5260_079330	pseudouridine synthase	*pumJ*	AVT42379.1	80/88
WT5260_079340	carboxylate-amine ligase	*pumK*	AVT42380.1	72/78
WT5260_079350	export	*pumL*	AVT42381.1	76/79
WT5260_079360	amide ligase	*pumM*	AVT42382.1	75/82
WT5260_079370	guanidinoacetic acid formation	*punN*	AVT42383.1	89/92
WT5260_079380	unknown	*pumO*	AVT42384.1	78/82

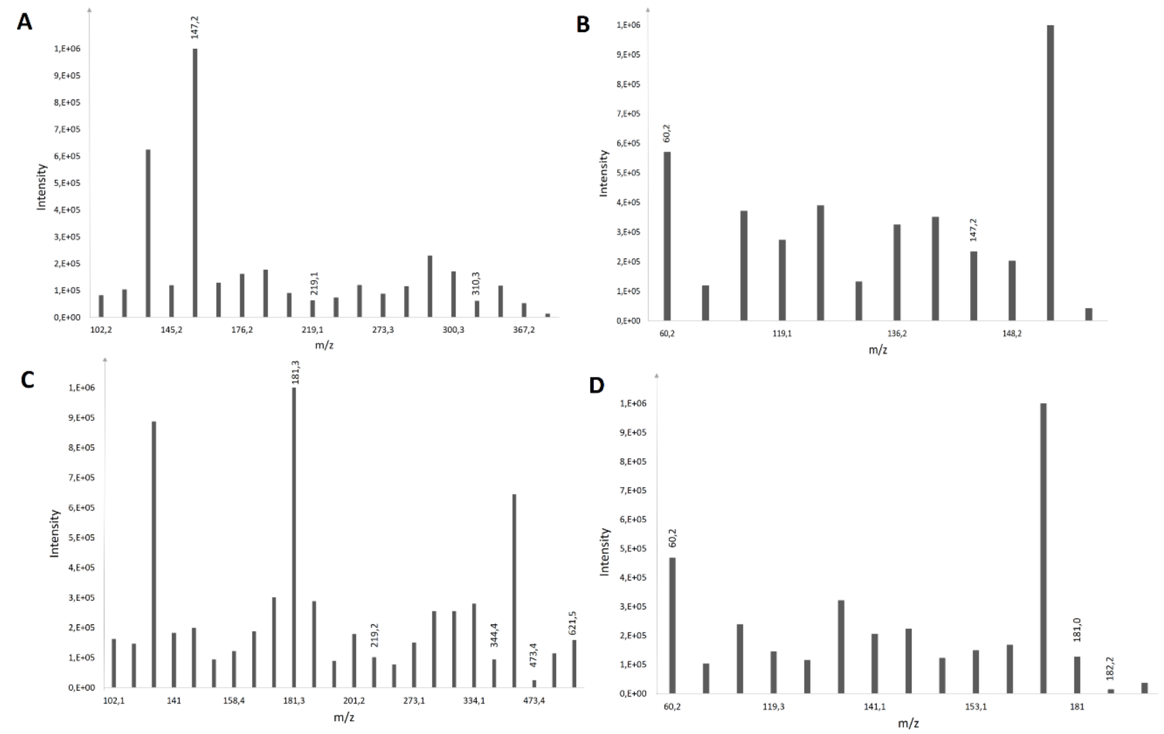

Figure S9: MS^2 spectra for **(A)** tyrobetaine, **(B)** tyrobetaine-2, **(C)** chlorotyrobetaine and **(D)** chlorotyrobetaine-2 produced by *S. rimosus* ATCC 10970 with representative peaks that correspond to already published MS^2 spectra published by Parkinson *et al*. (5).

References

1. Pikl Š, Rincón AFC, Slemc L, Goranovič D, Avbelj M, Gjuračić K, Sucipto H, Stare K, Baebler Š, Šala M, Guo M, Luzhetskyy A, Petković H, Magdevska V. 2021. Multiple copies of the oxytetracycline gene cluster in selected Streptomyces rimosus strains can provide significantly increased titers. Microbial Cell Factories 20.
2. Slemc L, Pikl Š, Petković H, Avbelj M. 2021. Molecular Biology Methods in Streptomyces rimosus, a Producer of OxytetracyclineMethods in Molecular Biology.
3. Shirling EB, Gottlieb D. 1966. Methods for characterization of Streptomyces species. International Journal of Systematic Bacteriology 16.
4. Sosio M, Gaspari E, Iorio M, Pessina S, Medema MH, Bernasconi A, Simone M, Maffioli SI, Ebright RH, Donadio S. 2018. Analysis of the Pseudouridimycin

Biosynthetic Pathway Provides Insights into the Formation of C-nucleoside Antibiotics. Cell Chemical Biology 25.

5. Parkinson EI, Tryon JH, Goering AW, Ju KS, McClure RA, Kemball JD, Zhukovsky S, Labeda DP, Thomson RJ, Kelleher NL, Metcalf WW. 2018. Discovery of the Tyrobetaine Natural Products and Their Biosynthetic Gene Cluster via Metabologenomics. ACS Chemical Biology 13.
